# Modeling Transmembrane Domain Dimers/Trimers of Plexin Receptors: Implications for Mechanisms of Signal Transmission across the Membrane

**DOI:** 10.1371/journal.pone.0121513

**Published:** 2015-04-02

**Authors:** Liqun Zhang, Anton Polyansky, Matthias Buck

**Affiliations:** 1 Department of Physiology and Biophysics, Case Western Reserve University, School of Medicine, 10900 Euclid Avenue, Cleveland, Ohio, 44106, United States of America; 2 Max F. Perutz Laboratories, Department of Structural and Computational Biology, University of Vienna, Campus Vienna Biocenter 5, Vienna, AT-1030, Austria; 3 M. M. Shemyakin and Yu. A. Ovchinnikov Institute of Bioorganic Chemistry, Russian Academy of Sciences, Moscow, 117997, Russia; 4 Departments of Neurosciences, Pharmacology, Case Comprehensive Cancer Center as well as Center for Proteomics and Bioinformatics, Case Western Reserve University, School of Medicine, 10900 Euclid Avenue, Cleveland, Ohio, 44106, United States of America; Aix-Marseille Université, FRANCE

## Abstract

Single-pass transmembrane (TM) receptors transmit signals across lipid bilayers by helix association or by configurational changes within preformed dimers. The structure determination for such TM regions is challenging and has mostly been accomplished by NMR spectroscopy. Recently, the computational prediction of TM dimer structures is becoming recognized for providing models, including alternate conformational states, which are important for receptor regulation. Here we pursued a strategy to predict helix oligomers that is based on packing considerations (using the PREDDIMER webserver) and is followed by a refinement of structures, utilizing microsecond all-atom molecular dynamics simulations. We applied this method to plexin TM receptors, a family of 9 human proteins, involved in the regulation of cell guidance and motility. The predicted models show that, overall, the preferences identified by PREDDIMER are preserved in the unrestrained simulations and that TM structures are likely to be diverse across the plexin family. Plexin-B1 and –B3 TM helices are regular and tend to associate, whereas plexin-A1, -A2, –A3, -A4, -C1 and –D1 contain sequence elements, such as poly-Glycine or aromatic residues that distort helix conformation and association. Plexin-B2 does not form stable dimers due to the presence of TM prolines. No experimental structural information on the TM region is available for these proteins, except for plexin-C1 dimeric and plexin-B1 – trimeric structures inferred from X-ray crystal structures of the intracellular regions. Plexin-B1 TM trimers utilize Ser and Thr sidechains for interhelical contacts. We also modeled the juxta-membrane (JM) region of plexin-C1 and plexin-B1 and show that it synergizes with the TM structures. The structure and dynamics of the JM region and TM-JM junction provide determinants for the distance and distribution of the intracellular domains, and for their binding partners relative to the membrane. The structures suggest experimental tests and will be useful for the interpretation of future studies.

## Introduction

**Fig 1:**
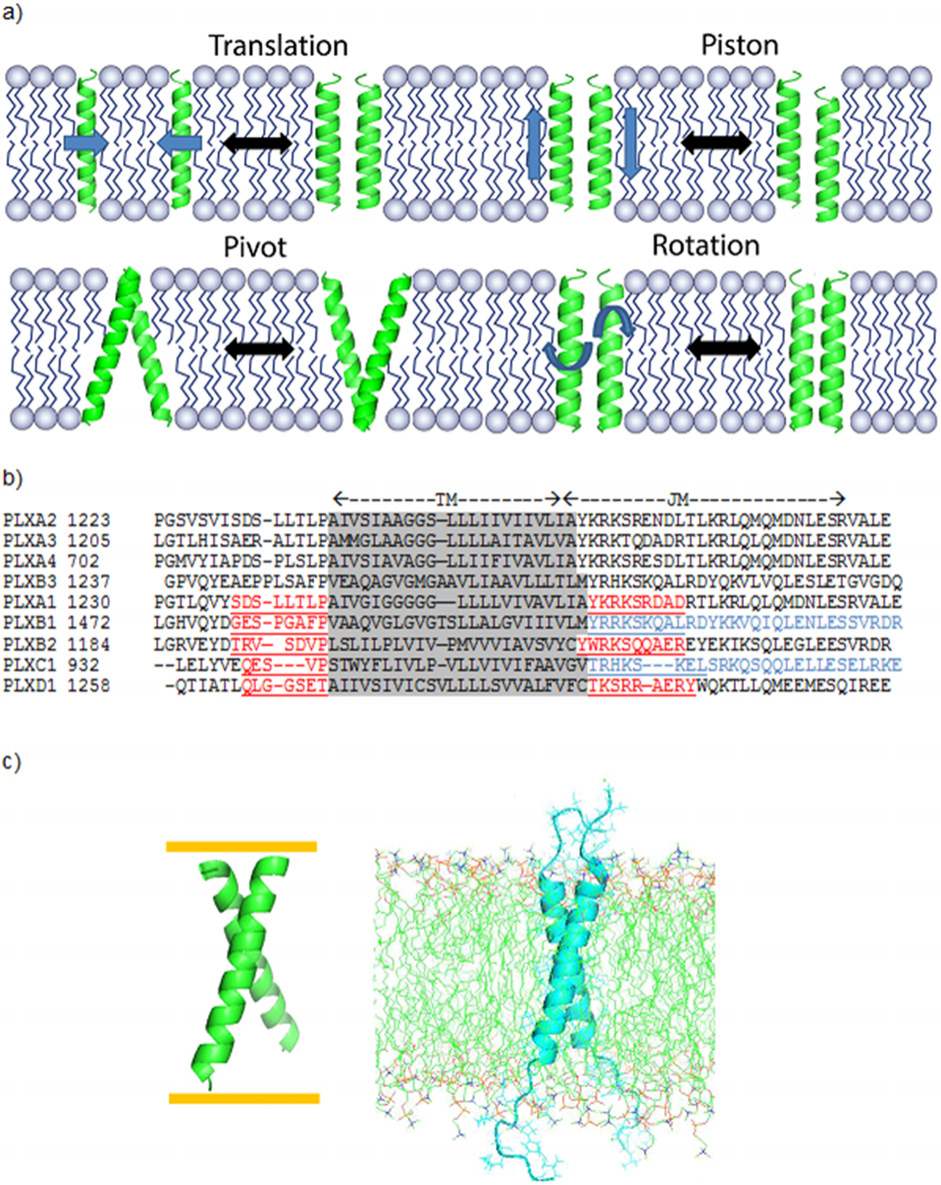
Models of plexin TM dimer receptor signaling, structure and sequence comparison. a). Modes for transmitting information across the cellular membrane in single pass TM receptors: Translation (monomer-dimer association); Piston (sliding of helices to change register); Pivot (change in interhelix crossing angles); and Rotation (change of helix interacting surfaces). b). Amino-acid sequence alignment of TM and TM proximal regions of all 9 human plexins: TM regions shaded grey, extra N- and C-terminal extensions underlined in red/blue for peptides, for which all-atom simulations were carried out, the juxta-membrane (JM) region is shown in blue for plexin-B1 and plexin-C1. The number after the plexin name corresponds to the first residue shown in the alignment. c). Comparison of plexin-B1 TM only peptide structure obtained from PREDDIMER (Left) and the same peptide with helix N- and C-terminal extensions (Right) embedded in lipid bilayer (structures of model b1.2 are shown); the peptide is shown as ribbon representation; the lipids are given in all-atom line representation on the right and the implicit bilayer is shown for the PREDDIMER prediction as orange lines on left.

How information is transmitted across cellular membranes remains a key problem in biology [1]. In the case of receptors that transverse the plasma membrane, ligand binding events on the outside are typically transmitted to the cytoplasm by configurational changes of the transmembrane (TM) regions, such as dimerization and/or conformational changes (for example in the orientation or position of TM helices relative to one another) (Fig. 1a) [2]. The study of membrane proteins remains challenging, especially for receptors that cross the membrane only once. Remarkably, no crystals of the helical TM regions of such receptors have been obtained/solved to date. TM domain structures for single-pass receptors, such as EGFR, ErbB2, EphA1, EphA2, and VEGFR2 (currently about 10 structures), have been derived by NMR spectroscopy or other biophysical techniques (e.g. [2,3]). Recently, molecular modeling and simulations play an increasing role for interpreting the experimental data [4,5,6,7,8]. Moreover, as the accuracy of reproducing the experimental structures increases, reliable predictions can be made. In this project, we advance on our previous study, which combined the prediction of helix contacts in TM dimers with extensive all-atom molecular dynamics (MD) [9]. Here we present predictions for the 9 members of the human plexin-family of TM receptors (plexin-A1-4, -B1-3, -C1 and -D1).

Plexins [10] are unique TM receptors in that they interact directly with small GTPases in diverse manners. This includes direct interactions with Rho GTPases and transient/catalytic interactions with Ras GTPases, as plexin functions as a GTPase Activating Protein (GAP) [10]. We have characterized the Rho GTPase Binding Domain (RBD) of plexin and developed a model, which posits a direct participation of Rho GTPases in the regulation of some of plexin’s functions [11,12,13]. Previously, it was shown that plexin signaling is outside-in (activation upon ligand binding outside) as well as inside-out (increased activation and ligand binding due to binding of certain Rho GTPases inside) [14,15]. While this mode of synergistic communication is seen in several other systems (e.g. for Integrins [16]), the molecular mechanism remains to be uncovered for plexins. Clearly, the TM region plays a key role, but given the low sequence similarity of this region across the 9 human proteins (Fig. 1b), the signaling mechanisms are likely to be diverse amongst members of the plexin family. For example, the typical GxxxG motifs, usually used for close helix-helix packing [17], are not well conserved between-or even within- plexin subfamilies. Thus, there is considerable interest to predict plexin TM helix dimer structures and to understand their configurational behavior.

We utilized a two-step approach: First, helix dimers were predicted *ab initio* based on helix packing considerations using the PREDDIMER server [18,19], a method that has been systematically benchmarked against known TM dimer structures. The best 3–7 predictions were then compared structurally across the family of 9 human plexins and 13 examples were chosen to cover the diversity of structures and subfamilies. Second, as an additional refinement, if not the testing step with respect to a state-of-the art all-atom forcefield, these structures were embedded in an explicit lipid bilayer and solvent (Fig. 1c) and equilibrated over a period of around 1.0 μs [9] on the MD optimized supercomputer Anton [20]. Nearly all of the predicted structures were stable and converged during these simulations. Thus, the *ab initio* predictions with PREDDIMER are relatively accurate. The diverse behavior of the TM helices across the plexin family is discussed. For plexin-B1, the best studied plexin to date, we also modeled the TM trimer and considered the role of part of the intracellular region, which immediately follows the TM segment: the so called juxta-membrane (JM) segment. Together with recent plexin-C1 dimer and plexin-B1 trimer structures of the intracellular region [21,22], we are able to make predictions concerning plexins’ configurational behavior and likely functional modes.

## Results

### 1. Comparison between PREDDIMER and CHARMM-forcefield all-atom μs-simulation refined TM structures

TM helix dimer structures were predicted *ab initio* for all 9 human plexins using the webserver PREDDIMER [19]. The full results are given in Table A in S1 File for the 26 best structures with packing scores (Fscor > 2.5). The PREDDIMER output was examined in terms of crossing angles, the location of the interface contact, and considered the rotation of the helices relatively to one another. Pairwise RMSD alignments were calculated and scaled for the extent of residue similarity between all of the 26 structures (see Table B in S1 File). Together, the helix geometric parameters suggested a grouping, with several additions to include at least two members of each subfamily. Thus, a diverse set of 13 structures was selected for refinement and testing. The parameters for these structures are given in Table 1, with comments on helix dimer configurations. Both right and left-handed crossed structures were selected and crossing angles range from 60^o^ to -55^o^, with several also near ± 10^o^; the latter indicating largely parallel helix configurations. Although all structures are homodimers, it should be noted that the predicted structures are not always symmetric. This is shown, for example, by the different helix rotation angles for model b1.7. The 13 models were then prepared for all atom molecular dynamics (MD) simulations as described in the Methods section.

**Table 1:** Initial configurations from PREDDIMER for TM dimer structures.

Model	Initial TM Dimer Structure				
	Fscor	crossing	A_rotation res.4, 11	B_rotation res 4, 11	Initial contacts
**a1.1**	3.0	-55.1	76.6, 68.4	93.8, 89.2	AIV**G**IGG**G**GG, GxxxG on both helices facing out, RH crossed, (final G at interface)
**a1.2**	2.9	-4.8	101.1, 84.4	79.4, 60.9	AIV**G**IGG**G**GG, GxxxG both out, parallel, (final G at interface), packed via LLLLVIV
**b1.1**	2.9	-37.0	45.6, -3.9	4.9, -17.8	Axxx**G**xxx**G** in, RH crossed; packed at **G**xxx**G**
**b1.2**	2.8	-52.1	131.2, 166.5	120.9, 175.4	AxxxGxxxG sidechains, opposite to interface, RH crossed using alternative **G**xxx**S** both in
**b1.3**	2.6	63.2	-131.8, -175.2	-154.5, 171.6	AxxxGxxxG sidechains both out; LH alternative **G**xxx**S** both in
**b1.6**	2.7	-25.1	103.4, 87.7	103.5, 87.7	AxxxGxxxG sidechains, both in, slight RH cross
**b1.7**	2.6	-15.4	105.0, 71.7	-24.1, -39.9	AxxxGxxxG one in—one out, parallel
**b2.1**	3.0	60.0	-145.0, 165.6	-145.0, 165.6	SLILP, P both in, LH cross
**b2.3**	2.6	-35.0	106.9, 60.6	106.9, 60.6	SLILP, P perpend. opposite to interface, RH cross
**c1.1**	3.4	4.9	-133.3, -93.1	-133.3, -93.1	PVLLV, P both out, parallel/slightly LH
**c1.2**	2.5	-50.0	-0.5, 30.0	6.2, 36.4	PVLLV, P perp. opposite to interface, RH
**d1.1**	3.0	55.0	-98.7, -150.0	-104.6, -155.7	SxxxCS; both in, LH cross at Cys9
**d1.2**	2.6	-5.1	-133.0, 176.9	-118.2, -169.1	SxxxCS; both in, parallel

**Fig 2:**
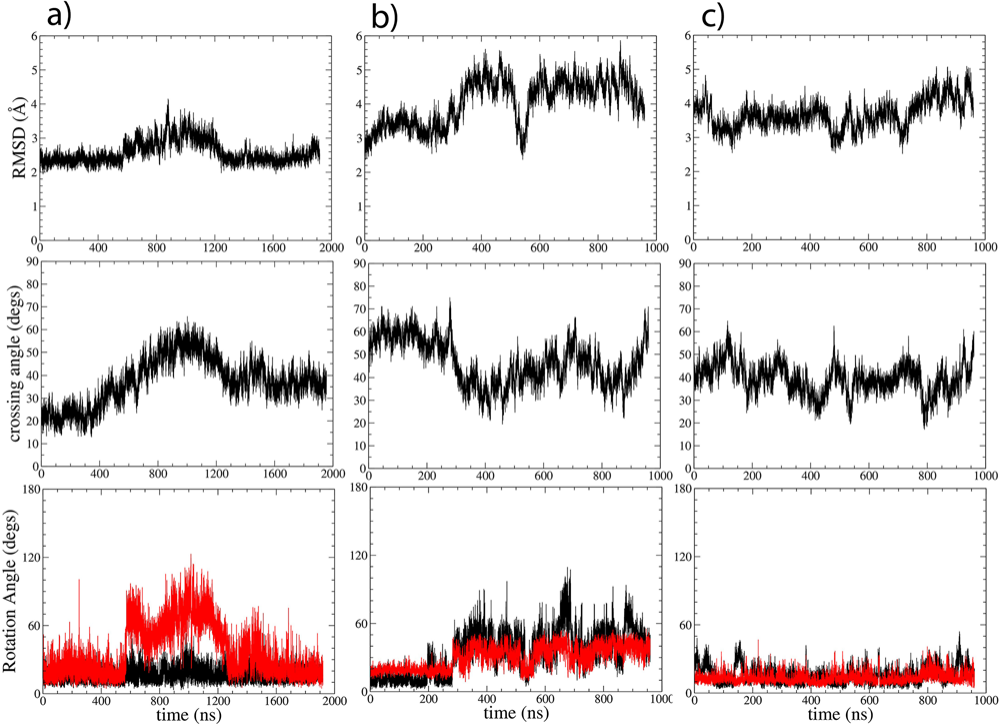
Structural variations of plexin-B1 models b1.1, b1.2 and b1.3 (panels a,b,c respectively) in MD simulations (TM + flanking residue simulations). RMSD (top), crossing (middle) and helix rotation angles (bottom panel), calculated as in [9]. Rotation angles for helix A in black, helix B in red. The data (see also methods and Table 2) suggest that 1 μs MD simulations are typically sufficient for the refinement. However, slower reversible changes are seen in simulation B1.1 which was continued to 2 μs. Standard deviations of RMSD and geometric parameters over the last 250 ns of the simulations were used to confirm equilibration.

**Fig 3:**
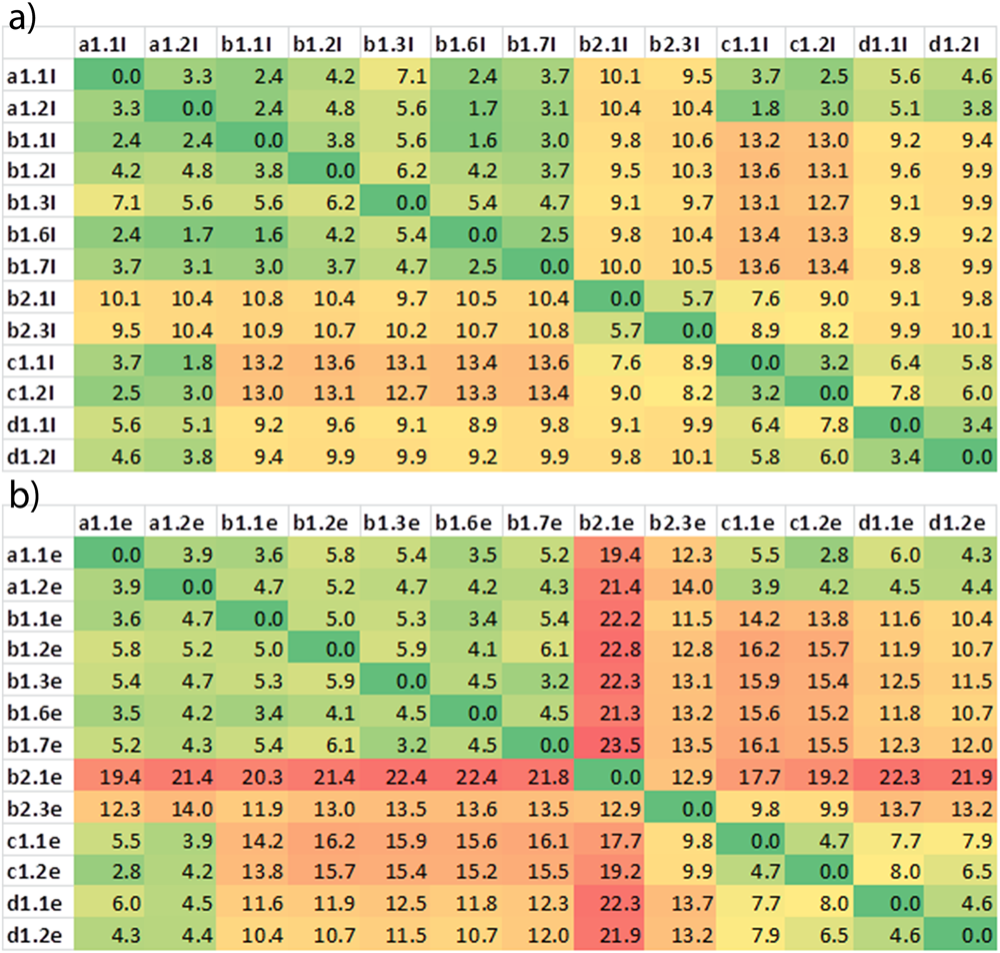
RMSD for initial and MD refined structures. a) RMSD values between initial (PREDDIMER predicted) structures across the subfamily of plexins chosen for further simulation. b) RMSD values between the structures (TM+extensions) after MD simulations, showing that the difference between the different subfamilies is essentially preserved.

Since regions immediately outside the hydrophobic TM segment can influence the helix dimer configuration (e.g. [5]), we extended the TM helix peptides by addition of up to 10 residues from the native human plexin sequences, both at the N- and C-termini (Fig. 1b,c). The structures were prepared as explained in the Methods section and simulated for 1.0 μs on Anton. One structure, b2.1, dissociated, while another structure, b2.3, showed a separation of the helices but contacts involving the both N- and C-terminal regions (the added residues) still holding the dimer loosely together. In order to verify the convergence of the simulations, plots of the evolution of the geometric parameters (RMSD to starting structure, helix crossing and rotation angles) were carefully examined for drift. While some of the structures fluctuate, drift was only apparent for crossing angles in Plexin-B1 model1, b1.1 and this simulation was continued to 2.0 μs (Fig. 2, also showing results for b1.2 and b1.3). We averaged the geometric parameters of the TM central regions for the last 250 ns of the simulations (see Methods). These values and the fluctuations around them are given in Table 2. Except for the b2.1 and b2.3 simulations the helix crossing and rotation angles are relatively well preserved from the PREDDIMER initial structures; however, the Fscor value is mostly decreased and becomes < 2.5 in 8 out of 13 simulations. By contrast, in 3 simulations b1.3, b1.6, d1.1, the packing is slight, and the packing in d1.1 is significantly improved over the initial structures. Large changes in packing coincide with larger changes in crossing angles (> ± 30^o^ in case of b1.7, b2.1, b2.3, d1.1, d1.2). Details of the final structures are given in Table 2, incl. the RMSD from the initial structures. Again, in correspondence with the geometric parameters, RMSD values are between 3.0–4.4 Å except for b1.7, b2.1 (which dissociated), and for d1.1 and d1.2. RMSD values less than 4.5 Å suggest that the structures are similar to those predicted *ab initio*, however there are slight adjustments in helix rotational angles (typically less than ±45^o^). Importantly, the relationship between the different plexin subfamilies identified in the *ab initio* predicted structures is largely preserved in the final, all-atom equilibrated structures of the helix dimers with N- and C-terminal extensions. Fig. 3a) gives the scaled pairwise RMSD values between the initial 13 plexin structures chosen for further refinement. Fig. 3b) gives the RMSD values between the final 13 plexin structures; reflecting that only b2.1 (which dissociates) changes with respect to the others.

**Table 2:** Final configurations from PREDDIMER for peptides with N- and C-terminal extensions after MD simulations.

**Model**	**Final with extensions**					
	Fscor	crossing	A_rotation, res. 4, 11	B_rotation, res. 4, 11	RMSD to initial (Å)	Comments on final structure
**a1.1**	4.4	-28.1	114.3,176.1	142.4,85.4	4.3	N-term. Distorted due to multiple G, esp. one helix badly kinked, mid. GI**G**G, with **G** interacting, slightly RH crossed
**a1.2**	1.5	9.8	168.6, -38.7	40.5, -123.2	4.0	N-term of both helices distorted due to multiple G, almost parallel, packed via C-term. **L**VI **V**AVL**I**, one helix shifted by close to 1 turn.
**b1.1**	2.9	-50.1	-155.2, -131.3	169.4, -141.8	3.0	Axxx**G**xxx**G** in, RH crossed; packed at **G**xxx**G** (for A) and **A**xxx**G** (for B); helix shift by 1 turn
**b1.2**	1.9	-53.4	113.4, 147.2	67.5, 117.8	4.0	AxxxGxxxG sidechains out, opposite to interface, RH crossed using alternative **G**xxx**S** both in—similar to initial but crossing angle slightly increased
**b1.3**	2.7	48.9	41.5, 78.5	65.7, 82.4	4.4	AxxxGxxxG sidechains out, LH crossing slightly decreased and helix shifted
**b1.6**	3.0	-49.1	-162.5, -171.0	168.3, 114.5	4.4	AxxxGxxxG both in, larger RH cross, little rotation
**b1.7**	0.8	39.9	-164.5, -124.6	-5.9, 36.0	6.0	AxxxGxxxG one in—one out, LH cross; large change in angle
**b2.1**	0.0	-55.0	102.7, -8.1	92.4, -21.7	14.8	Separated, after 350 ns
**b2.3**	0.7	2.0	-89.1, 19.7	103.6, -155.1	4.4	SxxxPxxxV, V both perpendicular; slightly separated, parallel; helices twisted and shifted
**c1.1**	2.0	6.8	26.5, 127.2	-81.5, -4.9	3.2	PVLLV, P one out, one perp.; N-term bulged due to WYF; parallel/slightly LH
**c1.2**	2.2	-31.2	-2.2, 39.7	5.2, 38.2	4.1	Helix bent at Pro10, PVLLV, P one in, one out, increased RH; separation at C-terminus; some unwinding at N-terminus
**d1.1**	2.6	21.0	-112.4, -138.5	-67.9, -126.6	4.5	SxxxCS; both in/perp. to interface, helixes shifted 1 up, 1 down, less LH crossing.
**d1.2**	1.3	-39.4	164.8, -124.9	28.2, 80.9	4.7	SxxxC both in/perp. to interface; RH crossing near C-term. Ser16; helix shift by ½ turn.

**Fig 4:**
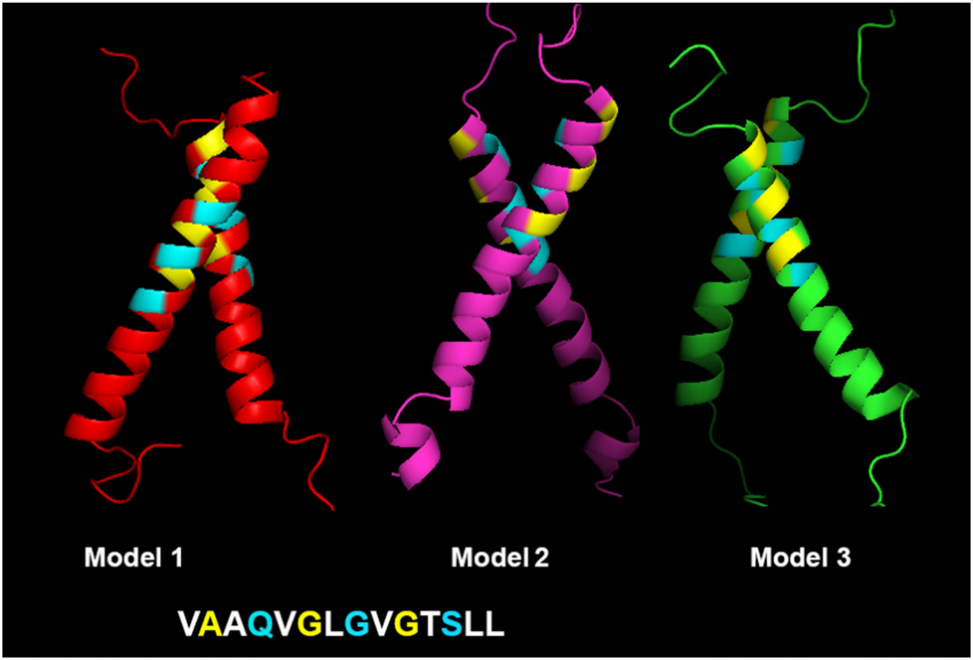
The final structures for plexin-B1 models b1.1, b1.2 and b1.3 after all-atom MD simulation. The geometric parameters for the final structures are shown in Fig. 3b. The first AxxxGxxxG dimerization motif is shown in yellow, the alternate motif, QxxxGxxxS in cyan. Models b1.1 and b1.2 are right-handed and b1.3 is left handed.

Looking at some of the simulations in greater detail, Figs. 2 and 4 show results for the MD refinement of the plexin-B1 models 1–3. In Fig. 2 the geometric parameters are plotted as a function of simulation time, illustrating that there are only few significant configurational fluctuations in crossing (Fig. 2b, middle panel) and relative rotation angles (Fig. 2a, lower panel). By 1.0 μs (and in case of b1.1 by 2.0 μs) the simulations are rather well converged in that the changes appear complete, giving us confidence that generally this time is sufficient to equilibrate the structures. In Fig. 4 the final structures for b1.1, b1.2 and b1.3 are shown, which includes both clockwise/right-handed (b1.1 and b1.2) and anti-clockwise/left-handed (b1.3) helix dimer structures. Here, the helices interact via two alternate sets of GxxxG-like motifs. The details of interactions stabilizing the structures, the extent of the observed fluctuations over the last 250 ns of the simulations and the likely functional consequences are discussed below.

### 2. Model for the plexin-B1 TM trimer

**Fig 5:**
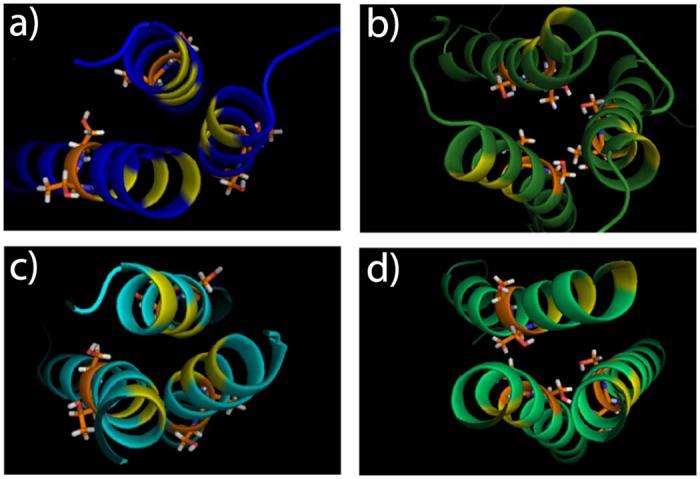
Models for plexin-B1 trimer TM region before (a,c) and after the simulations (b,d). Models with clockwise (a,c) and anti-clockwise (b,d) helix packing, looking from N-terminus into membrane. Helix A is on upper side, then B and C clockwise for clockwise, C and B for anti-clockwise. Ser and Thr sidechains are shown in stick (orange) and location of small residues (AxxxGxxxG) in the center of the TM region are indicated (in yellow).

The intracellular region of plexin-B1 has been crystallized in a trimeric state when bound to the small GTPase Rac1 [22]. It is important to test which configuration of the TM region would be compatible with a trimeric structure. Two TM trimer models, a left-hand/clockwise and a right-hand/anti-clockwise arrangement, were built and equilibrated for 1.0 μs on Anton. The initial and final structures are shown in Fig. 5 (and Fig. A in S1 File). Changes in the rotation angle for both clockwise and anti-clockwise helix trimer structures during the simulations are shown in Fig. B in S1 File. As can be seen, both the clockwise and anticlockwise structures are stable during this extensive simulation. There is a larger initial rotation at the contacting interface for helix C in the clockwise structure (left panel of Fig. B in S1 File) and this helix continues to fluctuate. Similar fluctuations are seen in helix A of the anti-clockwise structure.

Both structures have stable contacting interfaces, which are shown in Fig. 5. Particularly, Thr19 and Ser20 from the Plexin-B1 TM region make stable contacts in the trimers. The minimum distances of sidechain hydroxyls of Thr19/Ser20 that are located on neighboring helices, are plotted in Fig. C in S1 File showing, respectively, 1 and 2–3 relatively persistent Ser/Thr sidechain contacts (< 5.0 Å) in the clock-wise and anti-clockwise structures.

### 3. Model for the plexin-B1 TM-JM helix trimer

**Fig 6:**
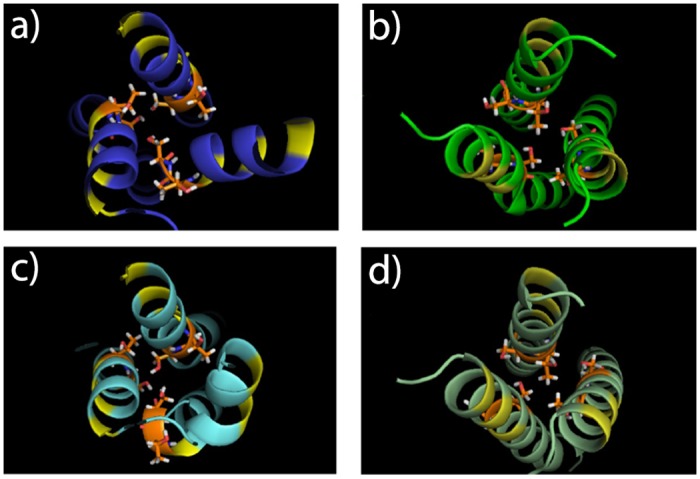
Models for plexin-B1 trimer TM region in the TM-JM models before (a,c on top panel) and after MD simulations (b,d on bottom panel). See legend for Fig. 4.

**Fig 7:**
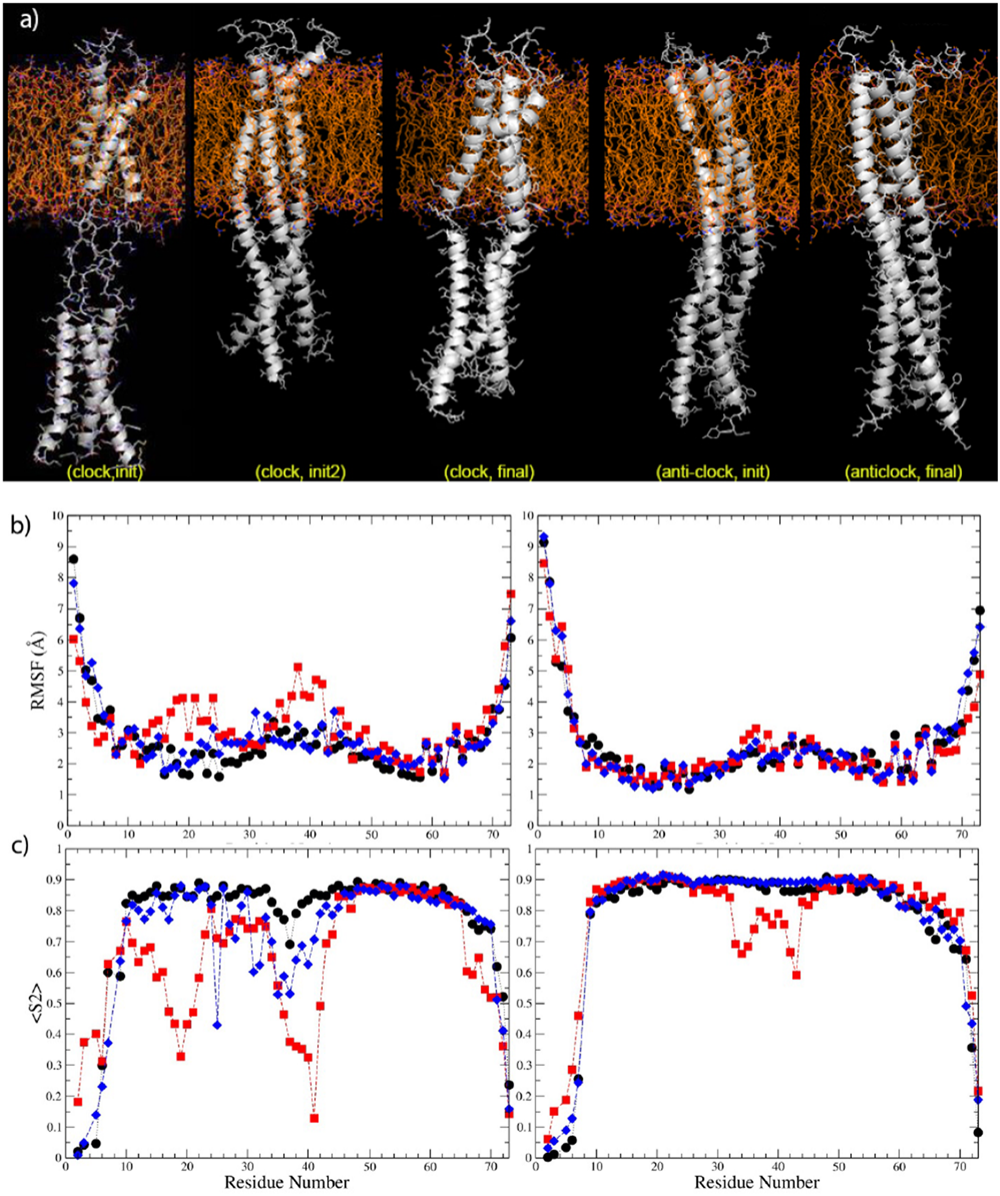
Model structures and dynamics of plexin-B1 TM-JM 9trimers. a) Two TM-JM trimer models for plexin-B1; structures are shown before MD refinement. Final structures are shown in Fig. B in S1 File. Left: extended clock-anticlock; Right: helix connected anticlock-anticlock. Distances of the JM C-terminal region from the membrane are plotted in Fig. F in S1 File. b) RMSF and c) <S^2^> for these plexin-B1 TM-JM trimers plotted as a function of sequence for the same simulations.

The juxta-membrane (JM) region, which connects the TM and intracellular domains was predicted to form a trimeric coiled coil. JM region was not visible in the X-ray structure of the trimer, but inferred from it [22]. Most of this region was, however seen in the X-ray structure of the plexin-B1 monomer [13]. Using the latter as a starting structure we built an anti-clockwise coiled-coil JM trimer as described in the Methods section and equilibrated it for 1.0 μs on Anton. The MD equilibrated clockwise or anti-clockwise TM trimer structures (described above) were then linked to this JM structure in several different ways: 1) as an extended connection in case of the TM clockwise/JM anti-clockwise arrangement, which was then restrained to become helical, 2) a bulged out-but otherwise irregular- connection for the TM anti-clock/JM anti-clock structure and finally, 3) the same with connections via helical (bent) structures, resulting in a total of 3 models. After equilibrating these configurations for 20 ns, it was clear that the structure started from model 2) showed very significant deviations from a helical structure and no further simulations were attempted. The structures started from model 1) and 3) were continued for 1.0 μs on Anton and had equilibrated. The initial and final structures are shown, partially in Fig. 6 and fully in Fig. 7a.

Does attachment of the JM region influence the configuration (and dynamics) of the TM regions? In order to address this question we calculated the RMSD values between the TM regions in the TM-only trimers, comparing initial and final structures and RMSD between the TM in the TM-only and in the TM-JM structures (Table 3). The results, also considering the fluctuations over the last 250 ns (Table C in S1 File) show that model2 (anticlockwise) TM segments and the whole TM-JM, are deviating from starting structures less than the clockwise structures (even in the case of TM only simulations). Slightly less deviation is seen in the JM region of the TM-clockwise structure, compared to the anticlockwise model. Joining the TM to the JM region reduces deviations in both TM-JM models, but especially in the anticlockwise TM compared to this TM’s initial structure.

**Table 3:** Plexin-B1 TM-JM trimer RMSD values comparing TM only and TM-JM structures before and after MD.

Comparison	Initial vs. Last in Model1 (clock)	Last vs. Last Model1, TM vs. TM in TM-JM	Initial vs. Last, in same Model2 (anticlock)	Last vs. Last, TM vs. TM in TM-JM Model2
a) TM-TM in TMonly	5.0	n/a	4.1	n/a
b.1) TM in TM-JM	3.8		2.1	
b.2) TM +/- JM		5.6		3.8
c) whole TM-JM	5.8	n/a	3.7	n/a
d) JM in TM-JM	2.5	n/a	3.1	n/a

RMSD values between the TM regions in the TM only trimers, comparing initial and final structures (a) and RMSD between the TM in the TM only and in the TM-JM structure, both with respect to their own initial (b.1) and with respect to each others final structures (b.2). We also compare the total deviation of the TM-JM structure (initial vs. final) (c) and similarly to a) compare the RMSD of the JM region between initial and final structures (d).

For the comparison of the dynamics, Root Mean Squared Fluctuation (RMSF) and order parameters (S^2^) were calculated. RMSF is a measure of the deviation of atomic positions from the trajectory average structure. S^2^, reflects the amplitude, here of NH bond fluctuations on the ps-ns timescale. S^2^ can also be derived from NMR relaxation measurements; thus this parameter is useful for future comparisons. The results are shown in Fig. 7b and 7c. Comparing the mainchain fluctuations of the TM clockwise and anti-clockwise structures, the anti-clockwise structure is more stable for all three helices, especially in the TM region. Fluctuations are seen in one of the helices in the TM-JM junction. The results for the corresponding TM trimers are shown in Fig. D in S1 File. Except for the clockwise TM-JM structure (above), the overall extent of fluctuations is similar in the TM part of the TM-only and TM-JM trimer simulations.

After considerable rotation of the helices during the initial model building of the TM-JM clockwise structure, both models show the TM region central Thr19 and Ser20 are localized into the interior of the 3-helix bundle (Fig. 6a, 6b). Several contacts between chains are relatively stable, shown by the minimal distances between the Ser20 and Thr19 residue pairs (Fig. E in S1 File). Similar to the plexin-B1 TM-only trimer models, the anti-clockwise TM-JM structure is more stable during the simulations than the structures involving the clockwise TM.

The distances between JM tail region (the last three residues) and the inner bilayer leaflet are shown in Fig. F in S1 File. The two plexin-B1 TM-JM trimers maintained a near constant distance throughout the simulations. The C-terminus of the TM-JM anti-clockwise/anti-clockwise structure is farther away from the lipid, and thus is less influenced by the interaction from lipids, and is more stable, not least because the helices are mostly regular including at the TM-JM junction. The final structures are shown in Fig. 7a. Running the Socket program [23], which can identify and analyze coiled-coil motifs within protein structures, both the plexin-B1 TM-JM helix trimer final structures are predicted to have coiled-coil structures. Such packing was not identified in the initial structures and developed during the all-atom MD simulations.

### 4. Plexin-C1 TM-JM helix dimer model

**Fig 8:**
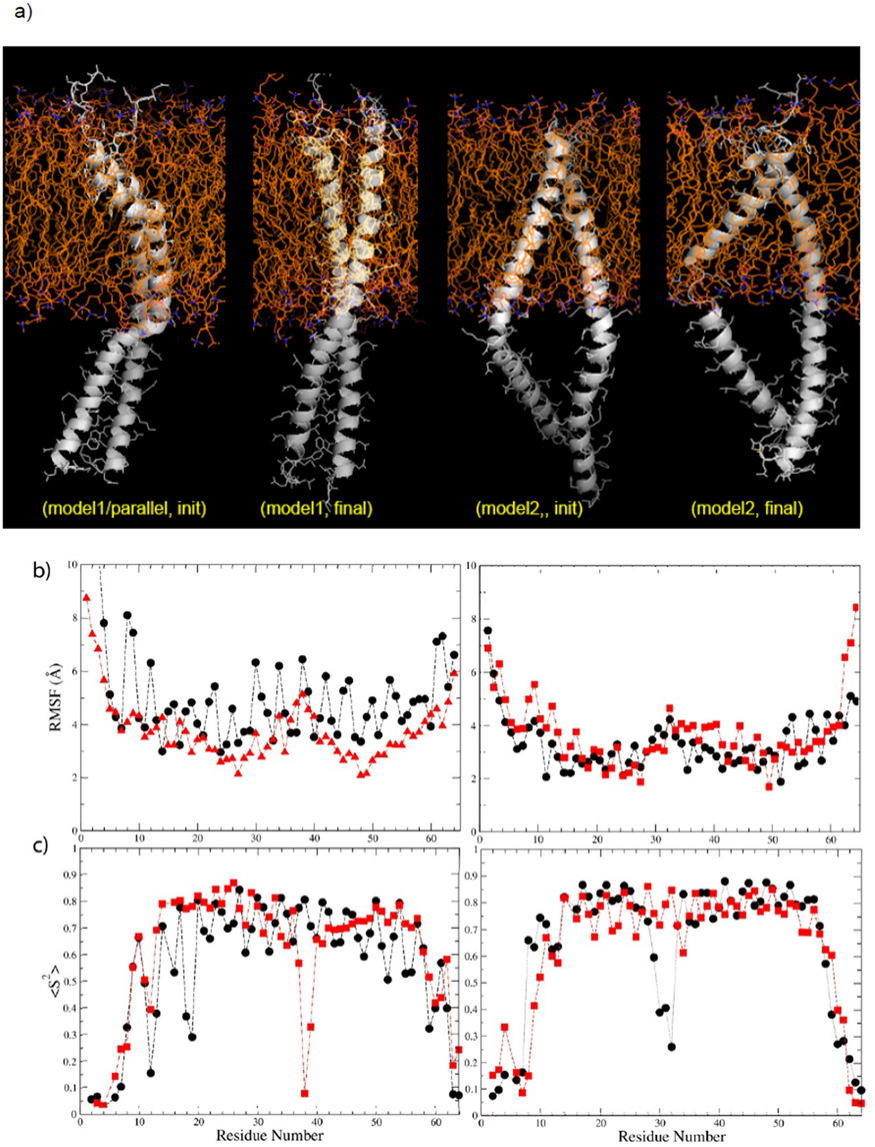
Model structures and dynamics of plexin-C1 TM-JM dimers. a). Two models (c1.2, left and c1.1, right) for the plexin-C1 TM-JM dimer connected by helical segments before 1 μs of MD. b) RMSF and c) <S^2^> both plotted as a function of sequence. Data for helix A in black, helix B in red. Also see S1 and S2 Movies.

In case of the plexin-C1 dimer [21], the crystal structure shows a coiled coil-like JM dimer (see Fig. G in S1 File for discussion and further analysis). The resolved part of the JM region needs to be extended to the membrane. It was modeled here, starting with the MD refined N- and C-terminally extended TM models, c1.1 and c1.2, which were then linked to the JM coiled-coil-like X-ray structure. The TM-JM junction was modeled in order to connect the TM and JM structures with continuing helices. However, we observed constraints during the modeling were imposed by the crossing angle of the TM region. These influence the packing of the junction, which is initially imperfect in one of the two helices in both structures (Fig. 8a).

Again, RMSD comparisons revealed the effect of adding the JM region on TM structures and dynamics (Table 4). The results show that the model1 (initial parallel) TM segments and the whole TM-JM, are deviating from the starting structures less than the model2 (right handed, RH) structures. Similarly less deviation is seen in the JM region of the parallel structure, compared to the RH model at the end of the trajectory. Surprisingly, joining the TM to the JM region increased deviations in both TM-JM models compared to their TMs initial structures. Similarly, comparing the final TM structures (with and without JM) shows that the deviations are slightly smaller than between the TM-JM initial structures. Interestingly, the difference between TM-JM models1 and 2 was reduced over the time-course of the simulations, possibly due to the fact the TM and JM regions of both models became more regular and better packed. We noted some of the issues of the final MD models c1.1 and c1.2, above—it appears that attaching the JM region fixed problems in the refined TM only models.

**Table 4:** Plexin-C1 TM-JM RMSD values comparing TM only and TM-JM structures before and after MD.

Comparison	Initial vs. Last Model1 (LH/parallel)	Last vs. Last Model1, TM vs. TM in TM-JM	Initial vs. Last, same Model2 (RH)	Last vs. Last, TM vs. TM in TM-JM Model2
a) TM-TM	3.4	n/a	3.9	n/a
b.1) TM in TM-JM	4.6		4.9	
b.2) TM +/- JM		4.1		4.3
c) TM-JM vs. TM-JM	6.9	n/a	9.3	n/a
d) JM only in TM-JM	2.3	n/a	3.2	n/a
JM only in Model1 vs. Model2 init/init. 6.8 last vs. last 5.2 whole Model1 vs. Model2 init/init. 8.6 last vs. last 6.1				

See Legend for Table 3.

In order to examine the extent of fluctuations across the TM-JM region, RMSF and S^2^ order parameters were calculated and are plotted in Fig. 8b and c, respectively. It is clear that although helices in model1 (built with c1.1) have a smaller crossing angle in the TM region/show more parallel helices, the fluctuations are greater than in model2 (c1.2), which had them crossed at a greater angle. More specifically, in model1 the helix A has a much larger structure fluctuation than helix B in both the TM and JM regions. At the same time, helix B was not continuous at first but has a bulge (see S1 Movie). Remarkably this bulge was fixed in the C-terminal JM region of helix B towards the end of the simulations. Although the bulge was likely to be responsible for some of the fluctuations, especially for the low S^2^ value (res. 38 of helix B), clearly there were other longer range packing defects also in the TM region and along the entire length of one side of helix A (seen as an oscillatory pattern). As a comparison, the RMSF and S^2^ results for TM only dimers were also calculated (Fig. H in S1 File). On average the RMSF values are 1 Å less than for the TM region in the TM-JM structure. Thus, the parallel TM dimer arrangement is not as stable, even when switching to the RH structure, crossed near the C-terminus of the TM region.

The model with the larger TM crossing angle (Fig. 8a right, built on c1.2) led to a separation of the N-terminal of JM/greater crossing of the C-terminal of JM coiled coil region and to a bulging out of the helical connecting structure, resulting in a break of one of the helices. The extended simulation of this structure did not change this local distortion. Except for this break of one helix in the TM-JM junction in model1 (res. 30–32 of helix A), which showed increased dynamics in the S^2^ analysis, the overall extent of the fluctuations is smaller compared to model1 (also see S2 Movie). A comparison with the TM only simulations for this model showed much greater fluctuations in helix A with an average RMSF of 4.0 Å for TM-only vs. 2.5 Å for TM in TM-JM. Helix B behaved similarly in both (RMSF of 2.5 Å). Thus, by contrast to model1 above, model2, with larger RH TM crossing angle experienced reduced fluctuations by being linked to the JM coiled-coil structure.

The distance between the C-terminal tail of the JM region and the inner bilayer leaflet was calculated for both model1 and -2 (Fig. I in S1 File). The C-terminus of model2 is on average 40 Å closer to the membrane compared to model1, reflecting the difference in crossing angle. However, at around 370 ns, the JM region of the model1 structure transiently came close to the lipid membrane (also shown in S1 Movie). Such interactions between the JM region and the lipid membrane can distort the JM helix structures, and influence the structure. By comparison no such large structural distortions were detected for TM-JM model2 during the all-atom MD simulation (shown in S2 Movie). The structure appeared to be overall more rigidly anchored in the lipid bilayer and the JM regions pointed consistently away from the membrane.

## Discussion

### 1. Consistency of PREDDIMER and all-atom molecular dynamics

Experimental structure determination for TM protein segments lags far behind the structures that are available for soluble protein domains [24,25]. Although recently there have been many structures solved for 7 TM receptor GPCRs and for other multi-transmembrane spanning channel and pore proteins, no crystal structures are so far available for single spanning TM proteins, such as plexin. The reasons for this are not clear, but it is possible that TM dimer and trimer structures are too flexible for crystallization/cannot be easily packed into a crystal lattice. Meanwhile, structures are available from other experimental techniques, chiefly from NMR but also structure predictions, and MD simulations of TM proteins are becoming increasingly reliable (e.g. [3,4,5,6,26,27]). We previously reported results on using an *ab initio* prediction strategy for TM helix dimers that involves an implicit representation for the lipid bilayer and for water. While allowing extensive configurational sampling [9], this method did not work well in our hands and appears to require additional input, such as symmetry [28] or at least helix-stabilizing restraints [29]. Several recent publications show that coarse grained simulations, and in one case computationally expensive 200 μs all-atom simulations, are able to obtain near experimental TM helix dimer structures from randomly placed TM helix monomers [30,31,8]. Here we tested a different approach, starting from structures that are predicted on the basis of helix packing modes, as implemented in the webserver PREDDIMER [19]. Similar to our previous study [9], which was validated with reference to two known TM helix dimer structures, we examined the predictions over an extensive time period (1 μs) by all-atom MD simulations. For this project we selected the family of plexin TM receptors. No experimental structures are known, but inferences for dimer and trimer TM structures could be made from recent crystal structures of the intracellular regions.

Computational resources did not allow us to carry out all-atom simulations for all the well-packed dimer structures that are predicted for the 9 human plexins. Thus, we considered groups of structures. The amino acid sequences of the TM regions of plexins are moderately well conserved within subfamilies as shown in Fig. 1b. These are the plexins-A1 to A4, which are similar in sequence but have a range of small residues near the N-terminus (A1 predicted as the most flexible, A4 as the least). Plexin-B1 and -B3 are close in sequence, but -B2 is different, having only one GxxxG-like motif near the N-terminus. Plexin-C1 and -D1 are also significantly different, with plexin–C1 having no clear GxxxG sequence. Thus, rather than grouping by sequence similarity, we grouped the predicted structures by geometric considerations and pairwise RMSD between all 26 PREDDIMER structures calculated for the plexin family. Indeed, with exception of plexin-B2, similar structures are predicted for members of the same subfamily as displayed in Fig. 3a).

A wide range of configurations of the two TM helices were predicted, with all 9 plexins showing some compatibility with TM parallel or crossed dimer arrangements (Fscor > 2.5, ranging from Fscor of 3.4 for plexin-C1 to 2.6 for -A3 and -B3). There is no initial preference of left- over right-handed crossings or more parallel arrangements from the PREDDIMER calculations. After grouping, 13 models were simulated for 1 μs on Anton. While this amount of time is usually too short to sample transitions between alternative states, we find (with exception of one case) that it is sufficient to allow an equilibration and thus a refinement of the *ab initio* models by use of the CHARMM27 forcefield [9]. If the structures are unstable, a separation or significant distortion of the helices is anticipated. Indeed one model for the plexin-B2 TM dimer experiences such larger configurational changes (discussed below). Examination of geometric parameters for the last 250 ns of the simulations shows that the simulations are equilibrated (i.e. RMSD < 0.5 Å for the central helix regions, crossing angles fluctuate within ±10^o^, and rotation angles within ±25^o^) as shown in Table D in S1 File. This is similar to deep minima seen in simulations begun with NMR derived structures [9] or with TM dimers that associated in all atom simulations [29]. In few cases larger fluctuations were observed, but these represent more shallow minima, rather than conformational drifts; nevertheless indicating that those predicted structures could be less well defined and TM dimers be less stable (e.g. models b1.7, c1.1). As with all classical MD simulations it is not possible to tell whether the structures are in a global energy minimum (e.g. in a 1000 μs simulation of BPTI some of the fluctuations only became apparent on the 100+ μs timescale) [32] and sampling transitions between different models is beyond the scope of the present work. To overcome the problem of potentially rugged energy landscapes, we used a different strategy, which we believe is efficient. We first predicted a number of possible dimer conformations available for a given sequences by the PREDDIMER algorithm and after that tested their persistence in the realistic lipid environment by microsecond MD simulations.

On average, the features of the predicted structures are maintained in the all-atom MD simulations, suggesting that the PREDDIMER predictions are overall reliable. It is likely that a lowered packing (Fscor) arises due to slight distortions of the helical structures in the MD simulations. Remarkably, the initial grouping is preserved when the structures were equilibrated in the extensive all-atom simulations (illustrated in terms of pairwise RMSDs, shown in Fig. 3a, 3b).

### 2. Biological Implication: A structural and functional diversity within the plexin family

#### Plexin-B2

The simulations suggest that not all of the plexin TM regions, by themselves, form strongly stable homodimers. Especially, plexin-B2 is an outlier, showing dissociation/low packing scores in the all-atom simulations. The TM region has little sequence similarity to those of plexin-B1 and -B3 and the refined structure is moderately similar to that of plexin-D1 (RMSD of 4.6 Å). It is less likely that plexin-B2 forms regular TM helix structures or such structures may dissociate for a number of reasons. Apart from a relatively high number of β-branched sidechains (11 out of 22), which tend to be helix destabilizing [33], it has two prolines (res.13 and 18) and no prominent GxxxG motif, except for SxxxP near the N-terminus. (The structure is also more extended since there are only 22 residues in -B2, compared to 24 in the-B1/B3 TM region). Another feature is several bulky sidechains, the YCYW sequence, at the TM region C-terminus, which as seen for plexin-C1, below, likely keeps helices apart. Plexin-B1 and especially plexin-B3 (only 6 β-branched sidechains) do not have such features. It would be tempting to infer that the unusual TM region of plexin-B2 influences the biological function and functional mechanisms of the receptor, which has been found to be considerably different compared to plexin-B1 and -B3 [34,35]. Other regions of the protein, for example the interacting region of the RBD domain with small GTPases, are also known to be substantially different [36]. Nevertheless, the RBD domain forms dimers in solution and also it is presumed that the extracellular ligand binding domain will form dimers, at least when bound to semaphorin ligand. Thus, the energetics of the TM region may synergize or be over-ridden by the oligomerization of the extra- and intracellular receptor regions.

#### Plexin-B1 (-B3)

Alternative helix packing motifs have been described for the EGFR and Ephrin receptor TM regions [30,36]; however these are non-overlapping GxxxG motifs near the N- and C-termini of the TM helices [37]. These motifs suggested a model of activation/inactivation due to a change in crossing angle. Recently, Zhang et al. [9] and others have described the partially overlapping/off-set GxxxG motifs that are compatible with a helix rotation to a different state; for ErbB1/B2 and EphA2 by approx.120 degrees on one of the helices. For plexin-B1, the offset in the GxxxG motifs is a shift of two rather than one residue, compared to the previous example. This results in an approximate 180 degree change of helix orientation as shown in Fig. 4. The functional implications are illustrated below with the plexin-C1 TM-JM dimer. In plexin-B3 a similarly shifted motif was seen, but with GxxxG rather than with AxxxGxxxG (with the N-terminal Ala being replaced by Glu in -B3). This change could destabilize such alternate configurations or would at least disfavor a helix parallel conformation as a possible intermediate. In the case of plexin-B1, none of the 5 configurations that were tested by simulations have very strong helix-helix packing as reflected in Fscor values after equilibration; the structure of the intracellular trimer as well as coiled-coil predictions for the JM region [38] suggests that a TM helical trimer is the more stable configuration, which is also indicated by the reduced fluctuations of the helixes in the trimer (esp. anticlockwise), compared to the dimer models.

### 3. Role of intra-membrane Ser/Thr in plexin-B1 trimers and in dimers

The TM central Thr-Ser motif is unique to plexin-B1. (Apart from isolated Ser or Thr in the TM central region of -A2 and -D1, other plexins do not have this motif. The Ser-Thr pair in the plexin-C1 TM sequence is at the N-terminus of the helix, but that does not form interactions either in any of the plexin–C1 TM or TM-JM model structures). In plexin-B1, no inter-helix Ser/Thr contacts are made in the 5 TM dimer structures that were examined by simulations (b1.1–1.3,1.6 and 1.7). Generally placing polar sidechains in the hydrophobic environment of the lipid bilayer is unfavorable but the Ser/Thr hydroxyl group can form a hydrogen bond to the carbonyl of the adjacent mainchain helix turn in TM helices. However, interhelical Ser-Thr sidechain contacts have also been observed in some structures (e.g. [39]). In plexin-B1 such contacts are persistent in both the TM only and TM-JM plexin-B1 models of the trimer over the course of the simulations, especially in the anti-clockwise structures. Mutating these residues may destabilize the trimer structure, compared to the dimeric forms, but trimers may still be stabilized by the JM regions, which are predicted by algorithms such as MultiCoil [40] for the B-family plexins [10,22].

### 4. Role of irregular structures: poly-Gly in plexin-As, helix shifts in plexin-D1 and -C1

#### The plexin-A family

Their TM regions are characterized by poly-glycine motifs in the membrane interior—see Fig. 1b, (GGGGG in case of -A1, GGG for -A3 and GG for -A2 and -A4). In the simulations of plexin-A1 we noticed a partial unfolding at this position, allowing a more extended structure (plexin-A family members only have 22 residues spanning the membrane, similar to plexin-B2 above). Alternatively, we saw for plexin-A1 that the helices were crossed at a significant angle with a different orientation of one or both of the N-terminal helix sections. The functional significance of this is not clear, except one may speculate that by itself the structures would be more flexible and may need additional support for the transmission of cell signals across the membrane. Indeed, plexin-A family members are thought to require Neuropilin-1 as a co-receptor. Very recently heterodimeric structures between plexin-A1 and Neuropilin-1 have been modeled using coarse grained simulations [31] and there is also experimental evidence for such heterodimers [41]. Poly-glycines near the N-terminal TM segments have been noticed in other proteins and are thought to be involved in cholesterol binding [42]. Particularly, such a possibility has been shown by the Sanders group for the amyloid precursor peptide [43]. It is not known whether cholesterol plays a role in the signaling behavior of plexin-A family members, especially in the regulation of plexin-A1 signaling.

#### Plexin-D1

As the sole member of the plexin-D family in humans, the structures appear to utilize a SxxxCS motif, with crossing either right or left-handed near the second Ser (residue16). The structures are relatively rigid, but are not symmetric/very regular since helices were shifted relative to one another in the membrane, consistent with helix dimer tilting, as well as crossing. Except for plexin-B1, -D1 has also the longest TM region with 24 residues spanning the membrane. Again, the structure of plexin-D1 may be stabilized by TM-region interactions with co-receptors, with Neuropilin-1 as a prominent candidate. However, functionally both neuropilin dependent and independent signaling mechanisms have been characterized in different settings [44].

#### Plexin-C1

The predicted structures for plexin-C1, the sole member of the C family, are considerably different from other plexins (e.g. see RMSD comparisons in Fig. 3a, 3b). The structures are not very regular—there is some helix bulging or unwinding at the N-terminus (C1.1 and C1.2 respectively) due to the sequence TWYF, which is all β-branched and the aromatics present large sidechains, preventing the helices to come close. However in other cases, it has been shown that single aromatics can stabilize TM helix dimers by stacking or cation-π interactions [45]. A TM central proline, furthermore can introduce a kink in the plexin-C1 helices (C1.2). As reflected in the Fscor values, these structures are only moderately well packed, due to the absence of a GxxxG-like motif, mostly in a near parallel manner. Nevertheless, the fluctuations in the geometric parameters are modest for model2 (larger for model1, which however, can be influenced by attachment of the intracellular membrane proximal region).

### 5. Effect of JM region on TM structures and their dynamics: example of plexin-C1 and -B1 TM-JM models

A critical question is how the TM segments, being more or less rigid helices, connect with the extra- and intracellular protein domains outside the lipid bilayer. The structures of these regions are typically responsible for ligand/adaptor protein binding in cell signaling. Changes, for example ligand induced dimerization, can be transmitted across the lipid bilayer most effectively if the junctions between the TM helix and the extra- and intracellular domains are relatively rigid. In this case there could be concerted large-scale changes in the orientation of the extra and intracellular domains with rigid connections with the TM segment facilitating the transmission of cellular signals as a mechanical event across the plasma membrane. For many TM receptors, such as the receptor tyrosine kinase superfamily, the connection between the TM and catalytic/kinase domain is not immediate but a JM segment presents a bridging region between the two domains, often also involved in a regulatory function (e.g. [30]).

In the case of plexins, the JM regions show a relatively well-conserved leucine-zipper/heptad repeat characteristic for coiled-coils as shown in Fig. 1b. Recently the crystal structure of plexin-C1 has been determined which, indeed, shows part of the JM region as a loose coiled-coil (see additional comments in Fig. G in S1 File). We, therefore, used the X-ray resolved part of the JM region of plexin-C1 and then sought to model the linker region to the two top models of the plexin-C1 TM region (one is nearly parallel and the other right-hand crossing, displayed in Fig. 8a). Similarly, a coiled-coiled trimer JM structure was modeled and attached to several models of the plexin-B1 TM trimer. The dynamics of the TM dimers and trimers were largely unchanged by attachment of the JM region over the course of the all-atom MD simulation. In fact, the TM structures allowed the JM structures to become more regular. Using the SOCKET program [31] to analyze the packing of the JM region (with a cutoff of 7.0 Å), we found coiled-coil structures in both the JM-regions of the final plexin-B1 structures after 1.0 μs of MD. While for plexin-C1 TM-JM dimers, the SOCKET program could not predict a coiled-coil structure in the JM region (even using a larger cutoff of 8.5 Å), nevertheless, here the JM region has a remarkable effect of regularizing the helices and helix contacts in the plexin-C1 TM region of RH crossed structures. These different scenarios illustrate that equilibrating multidomain structures, such as these TM-JM regions, with long-term all-atom MD simulations using the CHARMM forcefield, yields different effects of structures on each other (different levels of cooperative or competitive interactions). These features reflect on the different mechanical properties and thus suggest different cell signaling mechanisms of the systems.

### 6. Implications for the mechanism of signal transduction of plexins

Analysis of the flexibility of the TM-JM structures suggest that these are relatively rigid and are thus likely to help orient the intracellular domains of plexin relative to each other and the membrane. This is also apparent when distances between parts of the structures were examined.

**Fig 9:**
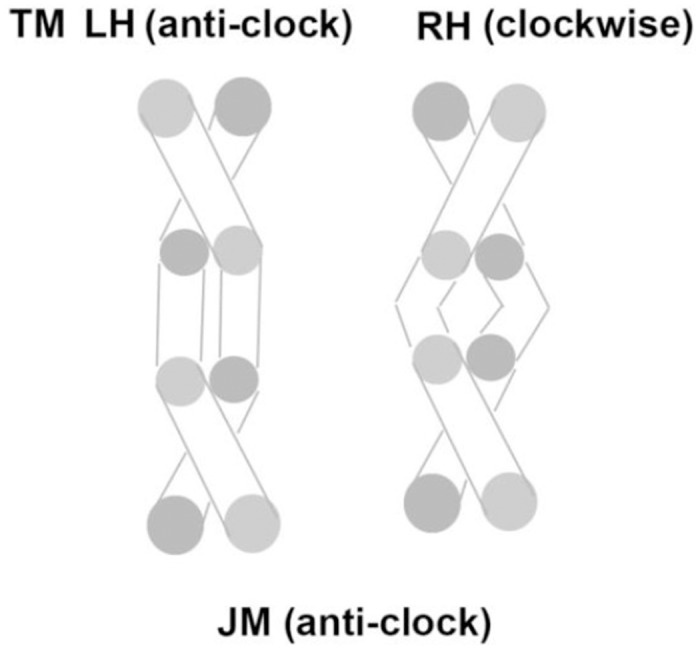
Schematic illustrating that structures with like-topology can be connected via straight segments, while unlike structures require some bending, either of irregular or of helical structures.

Firstly, one may consider the lateral distances and the rotation between the N- and C-termini of the TM helices as they enter/exit the membrane. In case of the TM trimer or largely parallel dimers, a modest amount of bulging would occur if unlike structures are connected. Nevertheless, the relative rotation of the TM (and especially JM) helices is similar in the plexin-B1 TM-JM trimer structures, suggesting that by contrast to the plexin-B1 dimers, the signaling mechanism would involve formation/dissociation of the TM-JM trimers, possibly via dimer intermediates. In the case of the two plexin-C1 TM-JM structures, the TM region differ more dramatically; in the parallel/slightly RH structure, the helices cross near Val21, with both sidechains at the interface. The bulged RH crossed structure, by contrast has the bulged helix rotated by ~ 180^o^ forming a back-to-back packing arrangement. In the JM coiled–coil, not only is the crossing angle different, but the bulged helix is also translated about 1 turn upwards and rotated by ~ 90^o^, relative to the parallel structure. This difference suggests a piston-like mechanism for TM signaling depicted in Fig. 1a, together with a rotation and separation of the intracellular plexin-C1 domains in the bulged/RH crossed structure. Overall, considering the number of contacts, the near parallel structure could be the more stable one by itself. However, larger lateral differences occur between parallel and crossed structures, as illustrated by plexin-C1. Here the distances between the TM-JM junctions of model1 and 2 are 11 Å and 23 Å, respectively—23 Å is sufficient to have 4.3 helical turns in an antiparallel helix coiled-coil arrangement for the JM region, similar to the example of EGFR [30]. Generally connecting like-structures (e.g. RH crossed helices with clockwise coiled-coils) results in more close-packed and less bulged structures than connecting unlike TM-JM structures as illustrated in Fig. 9.

Secondly, another consequence of the different TM-JM arrangements is the vertical distance between the membrane and the C-terminus of the JM region; in the results above the refined models show differences in this distance by up to 40 Å (see Fig. I in S1 File top panel). Membrane proximity is also known to be an important regulatory feature in another single TM helix receptor family, the EGFR receptors (e.g. ref. [46]). In the case of plexins, the primary binding partners are membrane anchored Rho and Ras GTPases, which associate with the RBD and GAP domains. The latter domains are also likely to influence plexin’s conformation near the membrane. This is the case for the dimer and trimer structures; especially the latter are expected to have more prescribed distances due to a locking of the three plexin units and GTPases into a ring type configuration [22]. Thus the orientations of the TM and JM segments, as well as the distances relative to the membrane, are likely to be important. For example, with an extended TM-JM connecting segment (as shown in Fig. 7a, left initial structure) there would be significant space between the plexin GAP domains and bound GTPases and the membrane, whereas with continuous helix linages—especially, with unlike structures, requiring bulged helices- this distance could be too short, at least for the binding of GTPases.

### 7. Summary and perspective

Our computational study for the first time comprehensively examined the TM region of the plexin receptor family. Predictions were made using TM helix packing, which were then tested/refined for the peptides in explicit solvent and lipid bilayer using all-atom simulations. We predict that the plexin family has diverse and alternate TM helix configurations and that intracellular JM coiled-coils likely synergize with the TM structures to create relatively rigid structures. These, in turn may be utilized for the regulation of plexin function. Now, guided by these models, experimental studies are needed to further validate these predictions.

## Materials and Methods

### 1. Structure prediction

For the predictions of the initial TM helix dimer structures for the 9 human plexins as shown in Fig. 1b), we used the webserver PREDDIMER [19], based upon the original algorithm that had been systematically benchmarked [18]. It should be noted that the server works only with the membrane embedded segment and N- and C-terminal extensions were added later (Fig. 1b, 1c). The membrane region limits of the sequence were defined by amino acid hydrophobicity as well as by sequence alignment. As extensions 5–8 and 9–10 amino acids were, respectively, added to the N- and C-termini of the 22–24 residue long TM segment.

Currently, no automated protocols are available for building TM trimer structures and we followed the procedure described elsewhere [47]. The models for a plexin-B1 TM trimer structure used either a left or a right handed crossed TM dimer and the third helix was manually docked (copy of helix 1) to give clockwise and anti-clockwise TM trimers, respectively. The structures were then equilibrated by molecular dynamics (MD) simulations and followed by 1.0 μs production runs on Anton.

In order to build a TM-JM helix dimer, we used the two PREDDIMER predicted and Anton-refined TM structures for human plexin-C1, c1.1 and c1.2, and fused these to part of the JM region (see Fig. 1b for sequence). Residues from Q553 to T584 were taken from the recently determined plexin-C1 Zebrafish dimeric structure (PDB ID: 4M8M) [21]. The missing residue sidechains, residues at the TM-JM junction as well as the sequence different from the human protein was rebuilt using MODELLER [48]. For the TM-JM trimer of plexin-B1, no structure for the trimeric JM region is available, but a large part of the JM region is seen bound to the plexin-B1 monomer (PDB ID: 3HM6, ref. [13]]. We took this structure and with reference to the plexin-C1 dimer, built sidechains that would be compatible with JM-GAP domain interactions. This suggested the orientation of the JM helices, which were then manually docked as a trimer and refined by simulation on Anton to 1 μs.

The initial structure after connecting the TM and JM regions is shown as an example for the TM-JM plexin-B1 trimer in the (clock, init) of Fig. 7a. In order to make the connection region to be helical, we ran short CHARMM simulations to relax the structure. The TM region and JM region were moved closer together to a distance that corresponds to the number of linking residues to be in a helical conformation. Then, we rebuilt the connection using MODELLER with a restraint that forced the connecting region to be helical. This turned nearly all the linker region into a helical structure. This structure, which is shown as (clock, init2) in Fig. 7a, as well as (anti-clock, init) for the anti-clockwise/anti-clockwise TM-JM plexin-B1 trimer was then embedded in the lipid bilayer/solvent system and used for the simulations. We built TM-JM structures with both a linking segment in an extended configuration, as well as in a helical conformation. In total, three models were continued with simulation refinement since the forth one showed a big bulge in the TM-JM connecting region. After insertion into explicit lipids and solvation, the peptides were equilibrated for around 20 ns using CHARMM. Based on deviations in the structures, we decided to continue with two models, one is a TM clockwise/right-handed crossing for plexin-B1 and, the other is an TM anti-clockwise/left-handed crossing TM model for the plexin-B1 trimer and an almost parallel helix dimer model for plexin-C1, which has a large crossing angle near the TM N- and JM- C-termini as shown in Fig. 8a.

### 2. MD simulations

The predictions were further refined/tested by all-atom MD simulations in explicit solvent and palmitoyloleoyl-phospatidyl-choline (POPC) lipid environment, following our previous work [9]. Briefly, the TM helix dimers to be refined were selected based on packing score of the predicted structures (Fscor > 2.5 and by a desire to have diversity in TM structures—see Table A in S1 File). The 13 structures selected were used as PREDDIMER output coordinates (see Table 1, 2) or one set of simulations; for a second set, these TM helices were extended by several residues at the N- and C-termini using MODELLER [48]. The polypeptide chain, the lipids and water molecules were simulated using the CHARMM36 [49] and TIP3P parameter sets. Structures were inserted into explicit POPC lipid bilayers (72 lipids/leaflet) using CHARMM-GUI [50] at a target area per lipid of 64.3 Å^2^ [51]. TIP3P water molecules were then added using CHARMM-GUI to yield a thickness (uniform above and below the bilayer) of no less than 15 Å from the farthest protein atom. Sodium and chloride ions were added to achieve neutral systems and further added to give a near-physiological ion concentration of 150 mM. Equilibration trajectories of the 2x13 systems were then generated in the NPAT ensemble with constant particle number N, at a normal pressure of 1 atm, and with the constant total surface area, obtained from CHARMM-GUI [50], and at a temperature of 310 K. Periodic boundary conditions were applied and electrostatic interactions utilized Particle Mesh Ewald with a real space cutoff of 12 Å. The same cutoff was used for the Lennard-Jones interactions. The SHAKE algorithm was applied to control the lengths of all bonds involving hydrogens and the integration time step was 2 fs. Using the CHARMM-GUI program, all systems were initially equilibrated for 300 ps before a further equilibration of a least 20 ns using CHARMM [52]. Then production runs were carried out to 1.0 μs on Anton.

### 3. Structure analysis

Root-mean-square-deviation (RMSD) values between helix configurations within and between plexin subfamilies were calculated in Pymol [53] and scaled according to the number of identical residues. Only the central region of TM helices (typically 14 residues, see below), which are always embedded inside the lipid membrane, were included in the RMSD calculation [9]. Specifically, RMSD obtained from alignment by ratio between the total number N of backbone (bb) atoms in both dimers and the number of atoms Na used for the alignment:

$${\text{RMSD}}_{\text{norm}}{\text{ = RMSD}}_{\text{align}}\text{ * }\left({\text{Nbb}}_{\text{diml}}{\text{ + Nbb}}_{\text{dim2}}\right)\text{ / }\left({\text{Nbb}}_{\text{dimla}}{\text{ + Nbb}}_{\text{dim2a}}\right)$$

The initial RMSD values were taken in Pymol; ignoring the option to eliminate outlying atoms.

Helix crossing angles were calculated as described in [9] using the CHARMM program. For the rotation angle calculations over the course of the simulation trajectories, we used the same method as [9] but calculated rotation angle data for individual residues rather than for the whole helices. This sets the rotation angle of the starting structure to zero and then evaluates the average but time specific rotation of the entire helices relative to this configuration. For the rotation angle shown in the tables in the main text and in the supplemental materials, a single structure was used as input to calculate the rotation angle. For helix rotation, residue 4 was chosen, about 1 turn of the helix into the membrane (a position that is represented in the 4 plexin-A family members, in -B1, and -B3 by either Gly, Ser or Gln), as well as residue 11, two further turns along (almost near the center of the 22–24 TM segments). (Unless the helices are distorted, these positions should be in alignment). The rotation angle of the 4th and, separately of the 11th residue from the N-terminus of the TM region relative to the vector of closest initial helix approach is calculated. For convenience all TM (and TM-JM) residues have been renumbered to start at 1 (numbering from first position of N-terminally extended sequence). RMSDs from the starting structures and geometric parameters were evaluated by visual inspection for drift and calculated over the last 250 ns of the simulations; averages and standard deviations are for the central region of the helices (typically res. 11–25) as given in Table C-E in S1 File. Models and simulations are referred to by plexin (e.g. A1) and then by model number, for example model1, to give a1.1.

Root-mean-square-fluctuation (RMSF) values were calculated using CHARMM which considered both the mainchain and sidehchain fluctuations. S^2^ for mainchain NH groups were calculated using the same method as in the previous work [54] using CHARMM based on the μs trajectories, but with a cut-off of 10 ns so that eventually the results may be compared to solution NMR measurements in micelles or bicells.

The distance between JM-tail and the membrane was also calculated using CHARMM, by calculating the closest distance from the center of mass of the last 3 residues in the JM domain to the closest heavy lipid head group atom in the lower bilayer.

## Supporting Information

S1 File **Fig. A**. Final structures for plexin-B1 TM trimer in clockwise orientation (Left) and anti-clockwise orientation (Right) after 1 μs MD simulation. **Fig. B**. MD fluctuation of the rotation angle of the two plexin-B1 TM trimer models. a) clockwise and b) anti-clockwise helix trimer. **Fig. C.** Minimum distances between OG/OG1 atoms on Thr/Ser residues on neighboring helices in 1 μs MD simulations with the initial plexin-B1 TM trimer structure started from the clockwise orientation (Left) and anti-clockwise orientation (Right). Helix A and C (red) form contacts during most of the simulation time in the clockwise orientation, while helices A and B (black), helix A and C (red) and helix B and C (green) form contacts in the anti-clockwise orientation. A more detailed analysis (not shown) reveals that in the clockwise structure between the A- and C-helices there is one Ser-Thr close (< 3.5Å) contact and two longer range Thr-Thr and Ser-Ser contacts (7–8Å). No interactions are seen in the other helix pairs. By contrast in the anticlockwise TM, there are close Thr-Thr and Thr-Ser contacts between the B and C helices, as well as a Thr-Ser contact between helices A and B. Between helices A-C and A-B there are longer range Ser-Thr and Thr-Thr contacts (~ 6–7Å). **Fig. D.** RMSF and <S^2^> of Plexin-B1 TM trimers. a) RMSF and b) <S^2^> of Plexin-B1 TM trimers as a function of sequence for clockwise orientation (Left) and anti-clockwise orientation (Right) trimers. Data for helix A in black circles, helix B in red squares, and helix C in blue diamonds. **Fig. E**. Minimum distances between atom OG/OG1 on Thr/Ser residues from neighboring helices for the plexin-B1 TM-JM trimer in clockwise direction (Left) and anti-clockwise direction (Right). A more detailed analysis (not shown) reveals that in the case of the clockwise TM refined model, there are two Ser-Ser (3–5 Å) (A-C and B-C) contacts and one far (~ 7 Å for A-B). Only one Thr-Ser is close (A to C). In the refined anticlockwise model there are one Ser-Ser (A-B at ~3.5 Å) and one Thr-Ser (A-C at ~3.5 Å) plus 5 longer range Thr-Ser/Thr-Thr contacts at approximately 6 Å. **Fig. F.** Distances between the C-terminal region of the JM trimer and the inner leaflet of POPC lipid bilayer for plexin-B1 TM-JM trimer in TM clockwise direction (Top) and TM anti-clockwise direction (Bottom). **Fig. G.** Structure comparison of the plexin-C1 TM dimer. Left) X-ray structure of zebrafish plexin-C1 with the GCN4 coiled coil region that has been added in order to crystallize this dimer (red/pink) (PDB ID: 4M8M [21]). The JM helices (green and cyan) are not strongly in contact and the coiling direction is clockwise, whereas the great majority of coiled-coil structures have an anti-clockwise twist [1,2]. Indeed, the sequence that was attached N-terminally to dimerize the plexin is derived from the GCN4 leucine zipper and shows anti-clockwise coiling. The observation that the native sequence is less strongly packed and has a slight clockwise twist suggests that the plexin JM region may not form a classical coiled-coil. Right) model of left-handed TM dimer (grey) and JM (yellow) helices with an irregular/extended junction. **Fig. H.** RMSF and <S^2^> of Plexin-C1 TM dimers. a) RMSF and b) <S^2^> of Plexin-C1 TM dimers as a function of sequence for the LH model dimer (Left) and RH model dimer (Right). Data for helix A in black circles, helix B in red squares. **Fig. I.** Average distances between C-terminal tails (C-alpha of three C-terminal residues) of the JM regions and the inner leaflet of POPC lipid bilayer for plexin-C1 TM-JM dimer in model1/LH model (top) and model2/RH model (Bottom) structures. **Table A**. Full table of PREDDIMER Predictions with Fscore > 2.5. **Table B.** Scaled RMSD between the central regions of initial TM structures from PREDDIMER. The structures with identifiers in red belong to the groups of 13 selected for further study. The remaining structures (black) are within an RMSD < 3.5 Å close to those selected as shown. **Table C.** RMSD, crossing angle, and rotation angle of helices for plexin-B1 TM trimer model1, TMtimer1+JMmodel1, TM trimer model2, TM trimer2+JMmodel2 after MD simulations. **Table D.** RMSD, crossing angle, and rotation angles of helices for TM+extension dimers after long-term simulations. **Table E.** RMSD, Crossing angle and rotation angle for plexin-C1 TM dimers and -C1 TM+JM dimers after MD simulations.
(DOC)

S1 Movie The plexin-C1 TM+JM dimer structure showed a structural distortion caused by its interaction with lipids during the long term MD simulations, with the initial structure starting from the model1 as shown in Fig. 8 Left. (MPG)

S2 Movie The plexin-C1 TM+JM dimer structure during the long term MD simulations, with the initial structure starting from the model2 as shown in the Fig. 8 Right. (MPG)

## References

[1] Hendrickson WA. Transduction of biochemical signals across cell membranes. Q Rev Biophys. 2005; 38(4): 321–30 ^.^ 16600054 10.1017/S0033583506004136

[2] Cymer F, Schneider D. Transmembrane helix-helix interactions involved in ErbB receptor signaling. Cell Adhesion Migration. 2010; 4: 299–312. 20212358 10.4161/cam.4.2.11191 PMC2900627

[3] Bocharov EV, Volynsky PE, Pavlov KV, Efremov RG, Arseniev AS. Structure elucidation of dimeric transmembrane domains of bitopic proteins. Cell Adh Migr.2010; 4(2): 284–98. 20421711 10.4161/cam.4.2.11930 PMC2900626

[4] Braun R, Engelman DM, Schulten K. Molecular Dynamics Simulations of Micelle Formation around Dimeric Glycophorin A Transmembrane Helices. Biophys J. 2004; 87: 754–63. 15298884 10.1529/biophysj.104.040279 PMC1304485

[5] Zhang J, Lazaridis T. Transmembrane helix association affinity can be modulated by flanking and noninterfacial residues. Biophys J. 2009; 96(11): 4418–27. 10.1016/j.bpj.2009.03.008 19486666 PMC2711494

[6] Stansfeld PJ, Sansom MS. Molecular simulation approaches to membrane proteins. Structure. 2011; 19: 1562–72. 10.1016/j.str.2011.10.002 22078556

[7] Im W, Jo S, Kim T. An ensemble dynamics approach to decipher solid-state NMR observables of membrane proteins. Biochim Biophys Acta. 2012;1818(2): 252–62. 10.1016/j.bbamem.2011.07.048 21851810

[8] Aci-Sèche S, Sawma P, Hubert P, Sturgis JN, Bagnard D, Jacob L, et al Transmembrane Recognition of the Semaphorin Co-Receptors Neuropilin 1 and Plexin A1: Coarse-Grained Simulations. Plos One. 2014; 10.1371/journal.pone.0097779 PMC4032258 24858828

[9] Zhang L, Sodt A, Venable R. M, Pastor R W, Buck M. Prediction and Refinement of ErbB1/B2 and EphA1 Transmembrane Dimers from Microsecond MD Simulations. Proteins. 2013; 81(3): 365–76. 10.1002/prot.24192 23042146 PMC3557542

[10] Hota P, Buck M. Thermodynamic characterization of two homologous protein complexes: Association of the semaphorin receptor plexin-B1 Rho GTPase binding domain with Rnd1 and active Rac1. Protein Science. 2009; 18: 1060–1071. 10.1002/pro.116 19388051 PMC2771308

[11] Tong Y, Hota PK, Hamaneh MB, Buck M. Insights into oncogenic mutations of plexin-B1 based on the solution structure of the Rho GTPase binding domain. Structure. 2008; 16(2): 246–58. 10.1016/j.str.2007.12.012 18275816 PMC2358926

[12] Tong Y, Chugha P, Hota PK, Alviani RS, Li M, Tempel W, et al Binding of Rac1, Rnd1, and RhoD to a novel Rho GTPase interaction motif destabilizes dimerization of the plexin-B1 effector domain. J Biol Chem. 2007; 282(51): 37215–24. 17916560 10.1074/jbc.M703800200 PMC2655321

[13] Tong Y, Hota PK, Penachioni JY, Hamaneh MB, Kim S, Alviani RS, et al Structure and function of the intracellular region of the plexin-b1 transmembrane receptor. J Biol Chem. 2009; 284(51): 35962–72. 10.1074/jbc.M109.056275 19843518 PMC2791024

[14] Vikis HG, Li W, He Z, Guan KL. The semaphorin receptor plexin-B1 specifically interacts with active Rac in a ligand-dependent manner. Proc Natl Acad Sci U S A. 2000; 97(23): 12457–62. 11035813 10.1073/pnas.220421797 PMC18785

[15] Vikis HG, Li W, Guan KL. The plexin-B1/Rac interaction inhibits PAK activation and enhances Sema4D ligand binding. Genes Dev. 2002; 16(7): 836–45. 11937491 10.1101/gad.966402 PMC186329

[16] Hu P, Luo BH. Integrin bi-directional signaling across the plasma membrane. J Cell Physiol. 2013; 228(2): 306–12. 10.1002/jcp.24154 22767296

[17] Russ WP, Engelman DM. The GxxxG motif: a framework for transmembrane helix-helix association. J Mol Biol. 2000; 296: 911–9. 10677291 10.1006/jmbi.1999.3489

[18] Polyansky AA, Volynsky PE, Efremov RG. Multistate organization of transmembrane helical protein dimers governed by the host membrane. J Am Chem Soc. 2012; 134(35): 14390–400. 10.1021/ja303483k 22889089

[19] Polyansky AA, Chugunov AO, Volynsky PE, Krylov NA, Nolde DE, Efremov RG. PREDDIMER: a web server for prediction of transmembrane helical dimers. Bioinformatics. 2014; 30(6): 889–90. 10.1093/bioinformatics/btt645 24202542

[20] Dror RO, Jensen MØ, Shaw DE. Elucidating membrane protein function through long-timescale molecular dynamics simulation. Conf Proc IEEE Eng Med Biol Soc. 2009; 2009: 2340–2. 10.1109/IEMBS.2009.5335057 19965181

[21] Wang Y, Pascoe HG, Brautigam CA, He H, Zhang X. Structural basis for activation and non-canonical catalysis of the Rap GTPase activating protein domain of plexin. Elife (Cambridge). 2013; 2: e01279 10.7554/eLife.01279 24137545 PMC3787391

[22] Bell CH, Aricescu AR, Jones EY, Siebold C. A dual binding mode for RhoGTPases in plexin signalling. PLoS Biol. 2011; 9(8): e1001134 10.1371/journal.pbio.1001134 21912513 PMC3166162

[23] Walshaw J, Woolfson DN. Socket: a program for identifying and analysing coiled-coil motifs within protein structures. J. Mol. Biol. 2001; 307: 1427–1450. 11292353 10.1006/jmbi.2001.4545

[24] Torres J, Stevens TJ, Samsó M. Membrane proteins: the 'Wild West' of structural biology. Trends Biochem Sci. 2003; 28(3): 137–44. 12633993 10.1016/S0968-0004(03)00026-4

[25] Garman EF. Developments in x-ray crystallographic structure determination of biological macromolecules. Science.2014; 343(6175): 1102–8. 10.1126/science.1247829 24604194

[26] Sayadi M, Tanizaki S, Feig M. Effect of membrane thickness on conformational sampling of phospholamban from computer simulations. Biophys J. 2010; 98(5): 805–14. 10.1016/j.bpj.2009.11.015 20197034 PMC2830431

[27] Jang H, Ma B, Woolf TB, Nussinov R. Interaction of protegrin-1 with lipid bilayers: membrane thinning effect. Biophys J. 2006; 91(8): 2848–59. 16861271 10.1529/biophysj.106.084046 PMC1578484

[28] Bu L, Im W, Brooks CL. Membrane assembly of simple helix homo-oligomers studied via molecular dynamics simulations. Biophys J. 2007; 92: 854–63. 17085501 10.1529/biophysj.106.095216 PMC1779983

[29] Li PC, Miyashita N, Im W, Ishido S, Sugita Y. Multidimensional umbrella sampling and replica-exchange molecular dynamics simulations for structure prediction of transmembrane helix dimers. J Comput Chem. 2014; 35:300–8. 10.1002/jcc.23494 24258786 PMC4083740

[30] Arkhipov A, Shan Y, Das R, Endres NF, Eastwood MP, Wemmer DE, et al Architecture and membrane interactions of the EGF receptor. Cell. 2013; 152(3): 557–69. 10.1016/j.cell.2012.12.030 23374350 PMC3680629

[31] Reddy T, Manrique S, Buyan A, Hall BA, Chetwynd A, Sansom MSP. Primary and secondary dimer interfaces of the fibroblast growth factor receptor 3 transmembrane domain: characterization via multiscale molecular dynamics simulations. Biochemistry. 2014; 53(2): 323–32. 10.1021/bi401576k 24397339 PMC4871223

[32] Shaw DE, Maragakis P, Lindorff-Larsen K, Piana S, Dror RO, Eastwood MP, et al Atomic-level characterization of the structural dynamics of proteins. Science. 2010; 330:341–6. 10.1126/science.1187409 20947758

[33] Adamian L, Nanda V, DeGrado WF, Liang J. Empirical lipid propensities of amino acid residues in multispan alpha helical membrane proteins. Proteins. 2005; 59(3): 496–509. 15789404 10.1002/prot.20456

[34] Roney KE, O'Connor BP, Wen H, Holl EK, Guthrie EH, Davis BK, et al Plexin-B2 negatively regulates macrophage motility, Rac, and Cdc42 activation. PLoS One. 2011; 6(9): e24795 10.1371/journal.pone.0024795 21966369 PMC3179467

[35] Azzarelli R, Pacary E, Garg R, Garcez P, van den Berg D, Riou P, et al An antagonistic interaction between PlexinB2 and Rnd3 controls RhoA activity and cortical neuron migration. Nat Commun. 2014; 5: 3405 10.1038/ncomms4405 24572910 PMC3939360

[36] Wang H, Hota PK, Tong Y, Li B, Shen L, Nedyalkova L, et al Structural basis of Rnd1 binding to plexin Rho GTPase binding domains (RBDs). J Biol Chem. 2011; 286(29):26093–106. 10.1074/jbc.M110.197053 21610070 PMC3138255

[37] Bocharov EV, Mayzel ML, Volynsky PE, Mineev KS, Tkach EN, Ermolyuk YS, et al Left-handed dimer of EphA2 transmembrane domain: Helix packing diversity among receptor tyrosine kinases. Biophys J. 2010; 98:881–9. 10.1016/j.bpj.2009.11.008 20197042 PMC2830432

[38] Trigg J, Gutwin K, Keating AE, Berger B. Multicoil2: predicting coiled coils and their oligomerization states from sequence in the twilight zone. PLoS One. 2011; 6(8): e23519 10.1371/journal.pone.0023519 21901122 PMC3162000

[39] Liu W, Eilers M, Patel AB, Smith SO. Helix packing moments reveal diversity and conservation in membrane protein structure. J Mol Biol. 2004; 337(3): 713–29. 15019789 10.1016/j.jmb.2004.02.001

[40] Wolf E, Kim PS, Berger B. MultiCoil: A Program for Predicting Two- and Three-Stranded Coiled Coils. Protein Science. 1997; 6: 1179–1189. 9194178 10.1002/pro.5560060606 PMC2143730

[41] Sawma P, Roth L, Blanchard C, Bagnard D, Crémel G, Bouveret E, et al Evidence for new homotypic and heterotypic interactions between transmembrane helices of proteins involved in receptor tyrosine kinase and neuropilin signaling. J Mol Biol. 2014; 10.1016/j.jmb.2014.10.007 25315821

[42] Koldsø H, Shorthouse D, Hélie J, Sansom SP Mark Lipid Clustering Correlates with Membrane Curvature as Revealed by Molecular Simulations of Complex Lipid Bilayers. 2014; 10.1371/journal.pcbi.1003911 25340788 PMC4207469

[43] Barrett PJ, Song Y, Van Horn WD, Hustedt EJ, Schafer JM, Hadziselimovic A, et al The amyloid precursor protein has a flexible transmembrane domain and binds cholesterol. Science. 2012; 336(6085): 1168–71. 10.1126/science.1219988 22654059 PMC3528355

[44] Chauvet S, Cohen S, Yoshida Y, Fekrane L, Livet J, Gayet O, et al Gating of Sema3E/PlexinD1 signaling by neuropilin-1 switches axonal repulsion to attraction during brain development. Neuron. 2007; 56(5): 807–22. 18054858 10.1016/j.neuron.2007.10.019 PMC2700040

[45] Kim T, Im W. Revisiting hydrophobic mismatch with free energy simulation studies of transmembrane helix tilt and rotation. Biophys J. 2010; 99:175–183. 10.1016/j.bpj.2010.04.015 20655845 PMC2895360

[46] Ziomkiewicz I, Loman A, Klement R, Fritsch C, Klymchenko AS, Bunt G, et al Dynamic conformational transitions of the EGF receptor in living mammalian cells determined by FRET and fluorescence lifetime imaging microscopy. Cytometry A. 2013; 83(9): 794–805. 10.1002/cyto.a.22311 23839800

[47] Kordyukova LV, Serebryakova MV, Polyansky AA, Kropotkina EA, Alexeevski AV, Veit M, et al Linker and/or transmembrane regions of influenza A/Group-1, A/Group-2, and type B virus hemagglutinins are packed differently within trimers. Biochim Biophys Acta. 2011; 1808(7): 1843–54. 10.1016/j.bbamem.2011.03.005 21420932

[48] Šali A, Blundell TL. Comparative protein modelling by satisfaction of spatial restraints. J. Mol. Biol. 1993; 234: 779–815. 8254673 10.1006/jmbi.1993.1626

[49] Huang J, MacKerell AD Jr. CHARMM36 all-atom additive protein force field: validation based on comparison to NMR data. J Comput Chem. 2013; 34(25): 2135–45. 10.1002/jcc.23354 23832629 PMC3800559

[50] Jo S, Lim JB, Klauda JB, Im W. CHARMM-GUI Membrane Builder for mixed bilayers and its application to yeast membranes. Biophys J. 2009; 97: 50–8. 10.1016/j.bpj.2009.04.013 19580743 PMC2711372

[51] Kučerka N, Nieh MP, Katsaras J. Fluid phase lipid areas and bilayer thicknesses of commonly used phosphatidylcholines as a function of temperature. Biochimica et Biophysica Acta (BBA)—Biomembranes. 2011; 1808(11): 2761–2771. 21819968 10.1016/j.bbamem.2011.07.022

[52] Brooks BR, Brooks CL 3rd, Mackerell AD Jr, Nilsson L, Petrella RJ, Roux B, et al CHARMM: the biomolecular simulation program. J Comput Chem. 2009; 30(10): 1545–614. 10.1002/jcc.21287 19444816 PMC2810661

[53] Schrödinger, LLC. The PyMOL Molecular Graphics System, Version 1.5.0.4

[54] Zerbetto M, Anderson R, Bouguet-Bonnet S, Rech M, Zhang L, Meirovitch E, Polimeno A, et al Analysis of 15N-1H NMR relaxation in proteins by a combined experimental and molecular dynamics simulation approach: Picosecond-nanosecond dynamics of the Rho GTPase binding domain of plexin-B1 in the dimeric state. J. Chem. Phys. B.2013; 117(1): 174–184. 10.1021/jp310142f PMC3556999 23214953

