# Supplementary material for: Modeling Transmembrane Domain Dimers/Trimers of Plexin Receptors: Implications for Mechanisms of Signal Transmission across the Membrane
Source: PLoS One. 2015 Apr 2;10(4):e0121513. doi: 10.1371/journal.pone.0121513 (PMC4383379; doi:10.1371/journal.pone.0121513)
Supplement: S1 File — Fig. A. Final structures for plexin-B1 TM trimer in clockwise orientation (Left) and anti-clockwise orientation (Right) after 1 μs MD simulation. Fig. B. MD fluctuation of the rotation angle of the two plexin-B1 TM trimer models. a) clockwise and b) anti-clockwise helix trimer. Fig. C. Minimum distances between OG/OG1 atoms on Thr/Ser residues on neighboring helices in 1 μs MD simulations with the initial plexin-B1 TM trimer structure started from the clockwise orientation (Left) and anti-clockwise orientation (Right). Helix A and C (red) form contacts during most of the simulation time in the clockwise orientation, while helices A and B (black), helix A and C (red) and helix B and C (green) form contacts in the anti-clockwise orientation. A more detailed analysis (not shown) reveals that in the clockwise structure between the A- and C-helices there is one Ser-Thr close (< 3.5Å) contact and two longer range Thr-Thr and Ser-Ser contacts (7–8Å). No interactions are seen in the other helix pairs. By contrast in the anticlockwise TM, there are close Thr-Thr and Thr-Ser contacts between the B and C helices, as well as a Thr-Ser contact between helices A and B. Between helices A-C and A-B there are longer range Ser-Thr and Thr-Thr contacts (~ 6–7Å). Fig. D. RMSF and of Plexin-B1 TM trimers. a) RMSF and b) of Plexin-B1 TM trimers as a function of sequence for clockwise orientation (Left) and anti-clockwise orientation (Right) trimers. Data for helix A in black circles, helix B in red squares, and helix C in blue diamonds. Fig. E. Minimum distances between atom OG/OG1 on Thr/Ser residues from neighboring helices for the plexin-B1 TM-JM trimer in clockwise direction (Left) and anti-clockwise direction (Right). A more detailed analysis (not shown) reveals that in the case of the clockwise TM refined model, there are two Ser-Ser (3–5 Å) (A-C and B-C) contacts and one far (~ 7 Å for A-B). Only one Thr-Ser is close (A to C). In the refined anticlockwise model there are [file pone.0121513.s001.doc]

## Modeling of Transmembrane Domain Dimers/Trimers for Plexin Receptors: Implications for Mechanisms of Signal Transmission across the Membrane

Liqun Zhang, Anton Polyansky, Matthias Buck

**Supplemental Materials**

**S1 Figures:**


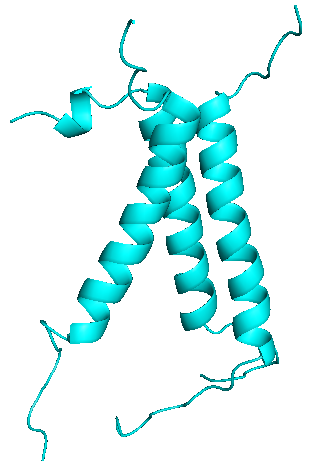

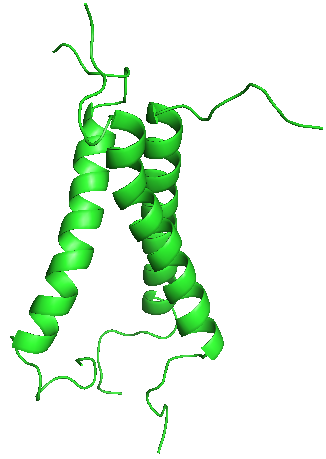


**Figure A**. **Final structures for plexin-B1 TM trimer in clockwise orientation (Left) and anti-clockwise orientation (Right) after 1 µs MD simulation.**


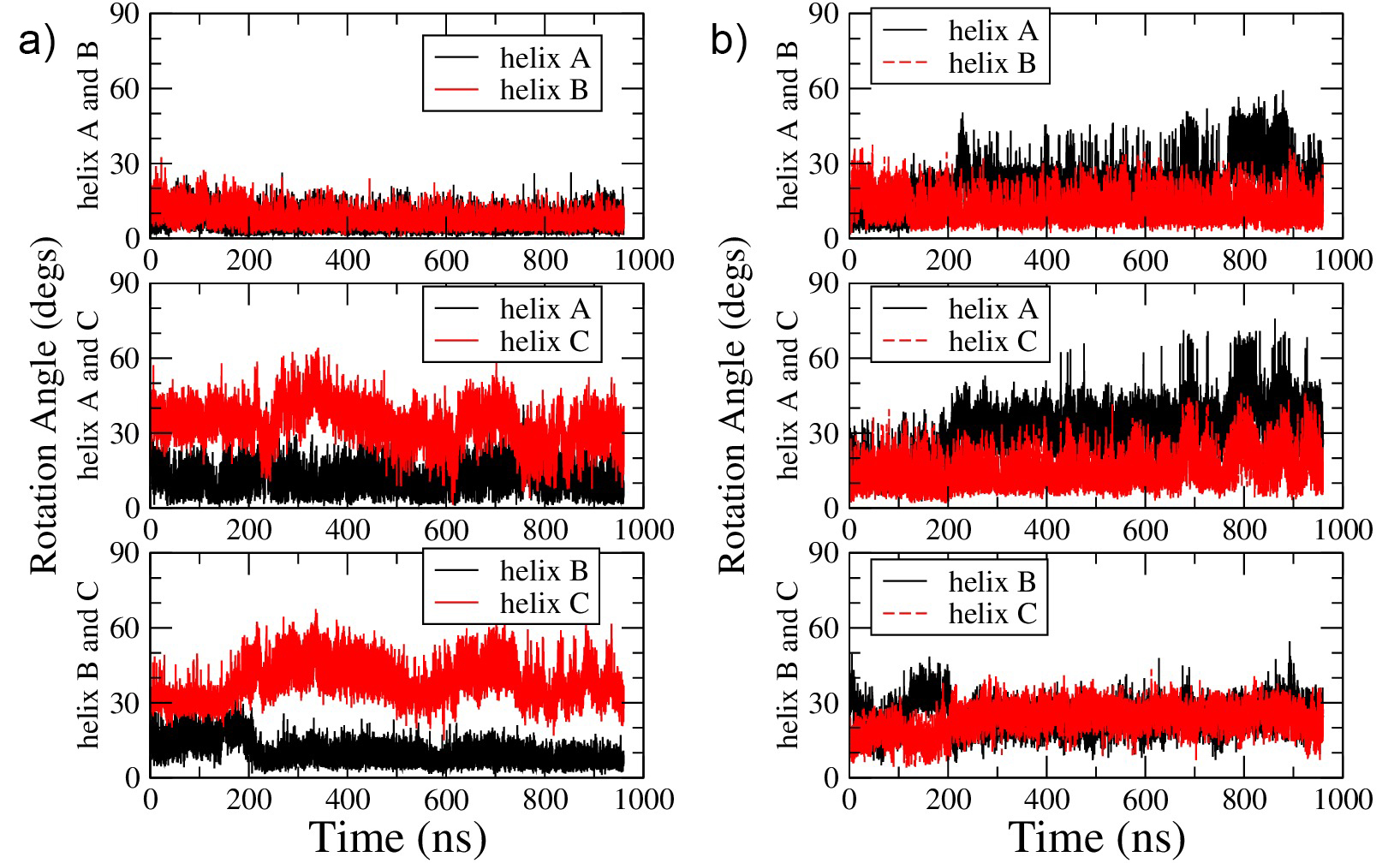


**Figure B**. **MD fluctuation of the rotation angle of the two plexin-B1 TM trimer models. a) clockwise and b) anti-clockwise helix trimer.**


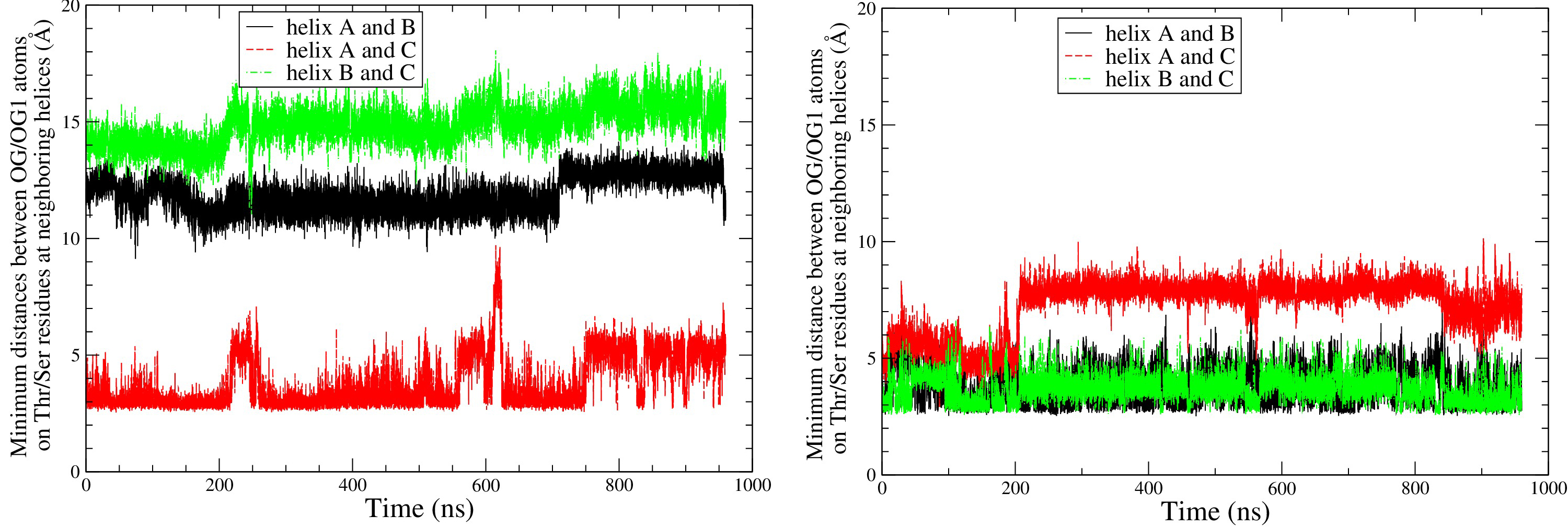


**Figure C. Minimum distances between OG/OG1 atoms on Thr/Ser residues on neighboring helices in 1 µs MD simulations with the initial plexin-B1 TM trimer structure started from the clockwise orientation (Left) and anti-clockwise orientation (Right)**. Helix A and C (red) form contacts during most of the simulation time in the clockwise orientation, while helices A and B (black), helix A and C (red) and helix B and C (green) form contacts in the anti-clockwise orientation. A more detailed analysis (not shown) reveals that in the clockwise structure between the A- and C-helices there is one Ser-Thr close (< 3.5Å) contact and two longer range Thr-Thr and Ser-Ser contacts (7-8Å). No interactions are seen in the other helix pairs. By contrast in the anticlockwise TM, there are close Thr-Thr and Thr-Ser contacts between the B and C helices, as well as a Thr-Ser contact between helices A and B. Between helices A-C and A-B there are longer range Ser-Thr and Thr-Thr contact (~ 6-7Å).


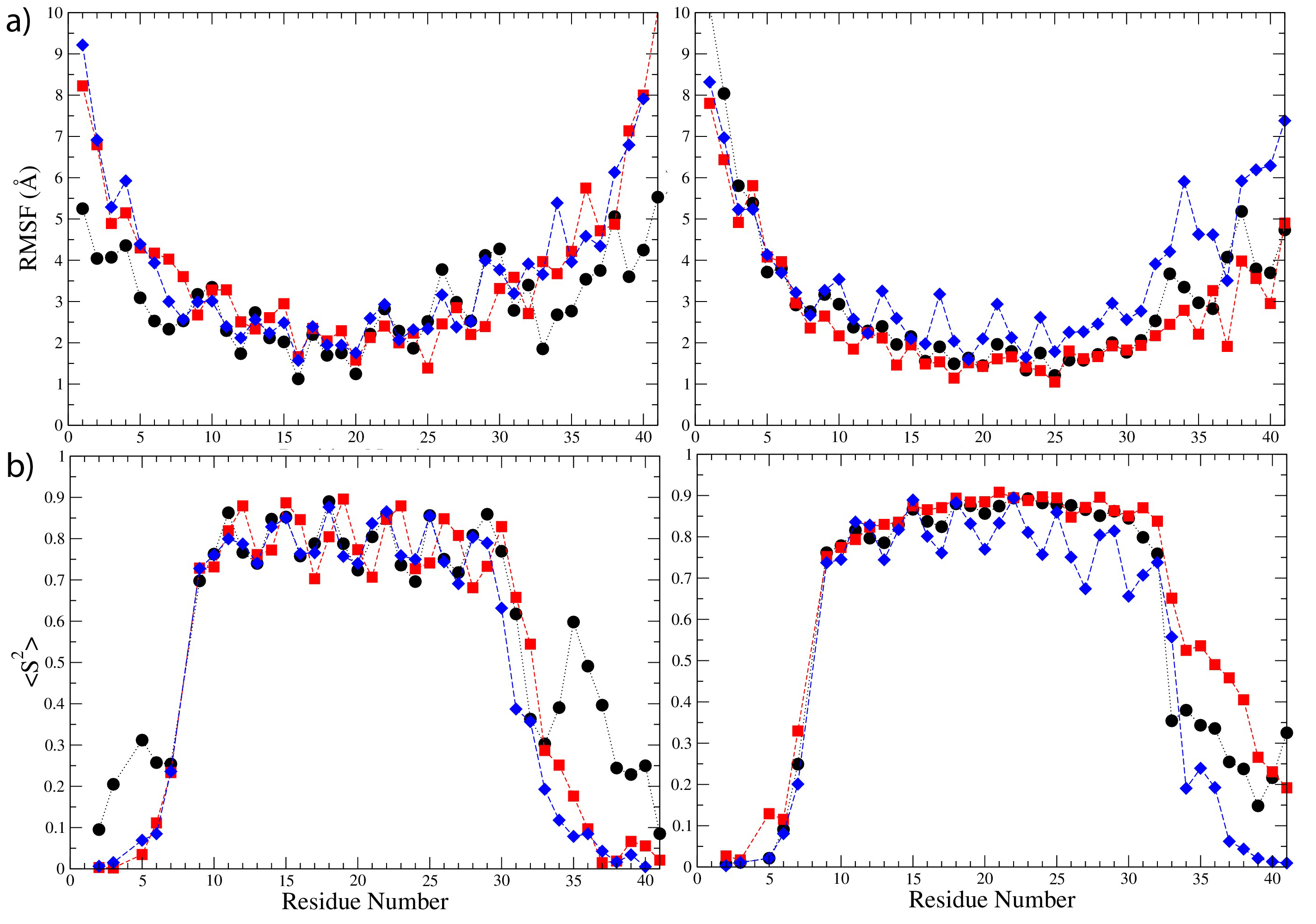


**Figure D. RMSF and <S^2^> of Plexin-B1 TM trimers as a function of sequence. a) RMSF and b) <S^2^> of Plexin-B1 TM trimers as a function of sequence for clockwise orientation (Left) and anti-clockwise orientation (Right) trimers. Data for helix A in black circles, helix B in red squares, and helix C in blue diamonds.**

**
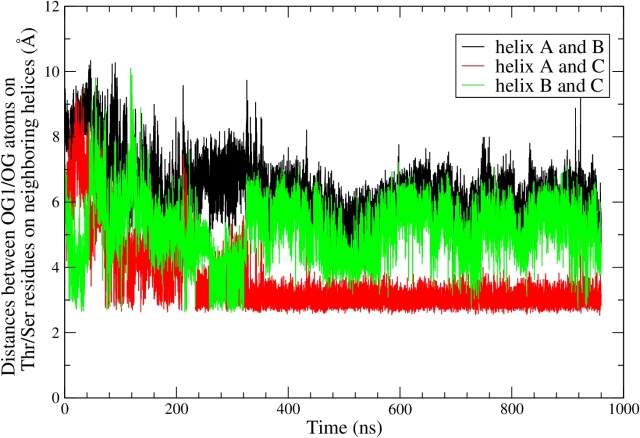

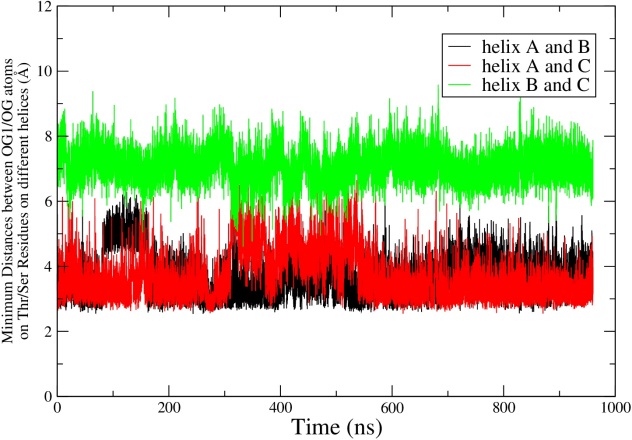
**

**Figure E**. **Minimum distances between atom OG/OG1 on Thr/Ser residues from neighboring helices for the plexin-B1 TM-JM trimer in clockwise direction (Left) and anti-clockwise direction (Right).** A more detailed analysis (not shown) reveals that in the case of the clockwise TM refined model, there are two Ser-Ser (3-5 Å) (A-C and B-C) contacts and one far (~ 7 Å for A-B). Only one Thr-Ser is close (A to C). In the refined anticlockwise model there are one Ser-Ser (A-B at ~3.5 Å) and one Thr-Ser (A -C at ~3.5 Å) plus 5 longer range Thr-Ser/Thr-Thr contacts at approximately 6 Å.

*
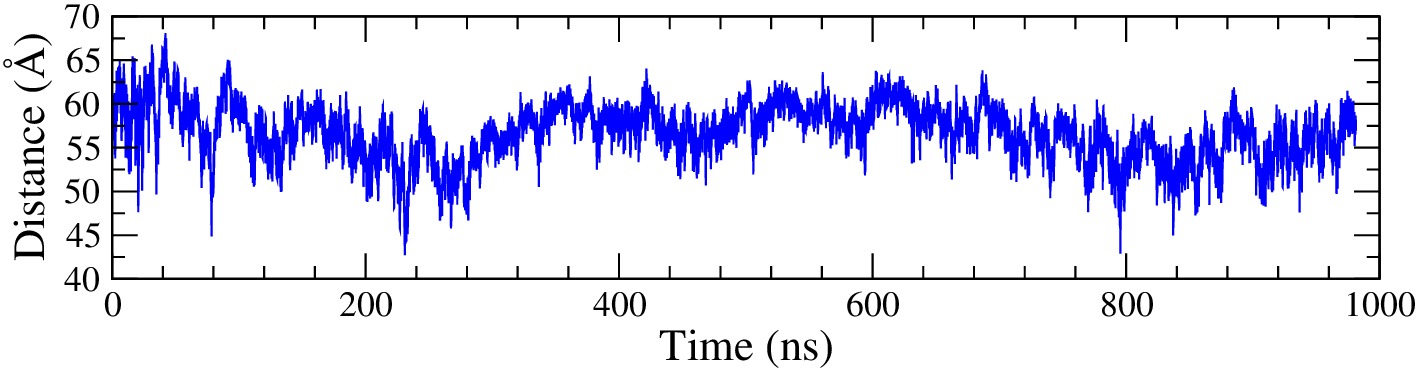
*

**
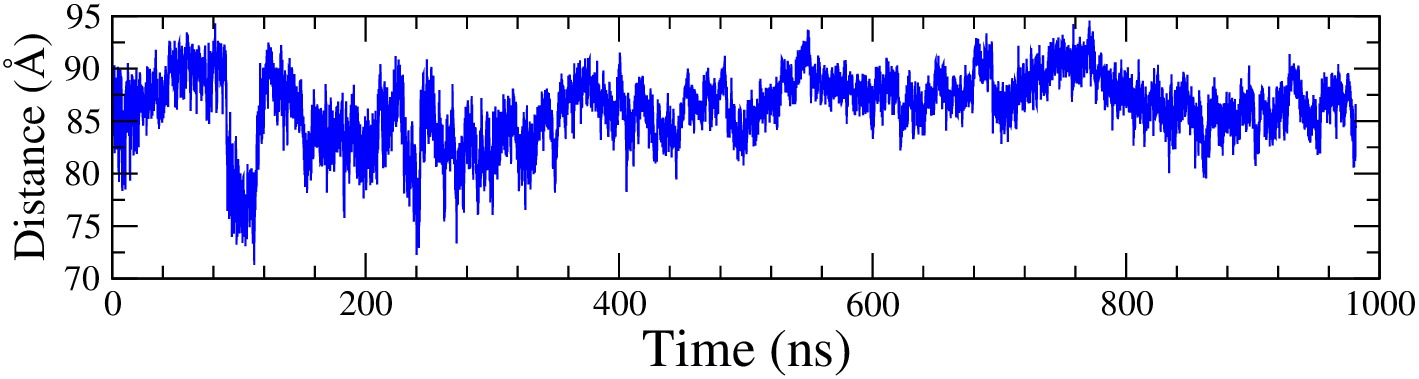
Figure F.** **Distances between the C-terminal region of the JM trimer and the inner leaflet of POPC lipid bilayer for plexin-B1 TM-JM trimer in TM clockwise direction (Top) and TM anti-clockwise direction (Bottom).**


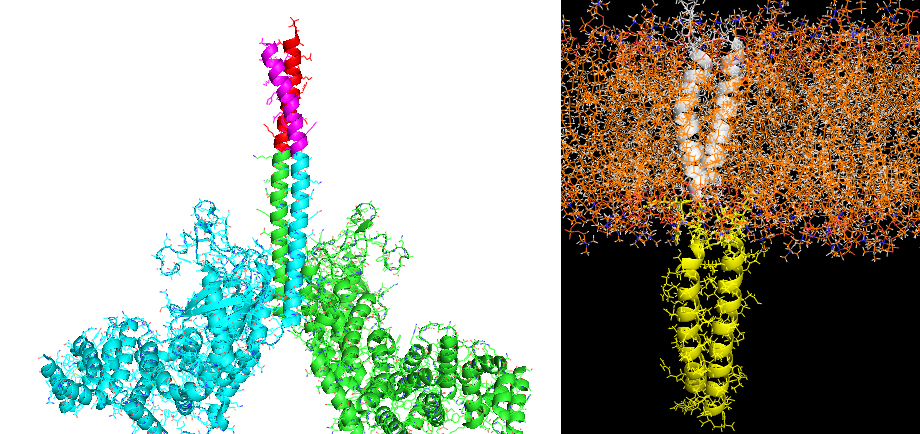


**Figure G. Structure comparison of the plexin-C1 TM dimer**. Left) X-ray structure of zebrafish plexin-C1 with the GCN4 coiled coil region that has been added in order to crystallize this dimer (red/pink) (PDB ID: 4M8M [21]). The JM helices (green and cyan) are not strongly in contact and the coiling direction is clockwise, whereas the great majority of coiled-coil structures have an anti-clockwise twist [1,2]. Indeed, the sequence that is attached N-terminally to dimerize the plexin is derived from the GCN4 leucine zipper and shows anti-clockwise coiling. The observation that the native sequence is less strongly packed and has a slight clockwise twist suggests that the plexin JM region may not form a classical coiled-coil. Right) model of left-handed TM dimer (grey) and JM (yellow) helices with an irregular/extended junction.

1. Chothia C, Levitt M, Richardson D (1981) [Helix to helix packing in proteins.](http://www.ncbi.nlm.nih.gov/pubmed/7265198) J Mol Biol. 145(1): 215-50.

2. Walshaw J, Woolfson DN (2001) [Socket: a program for identifying and analysing coiled-coil motifs within protein structures.](http://www.ncbi.nlm.nih.gov/pubmed/11292353) J Mol Biol. 307(5): 1427-50.

**
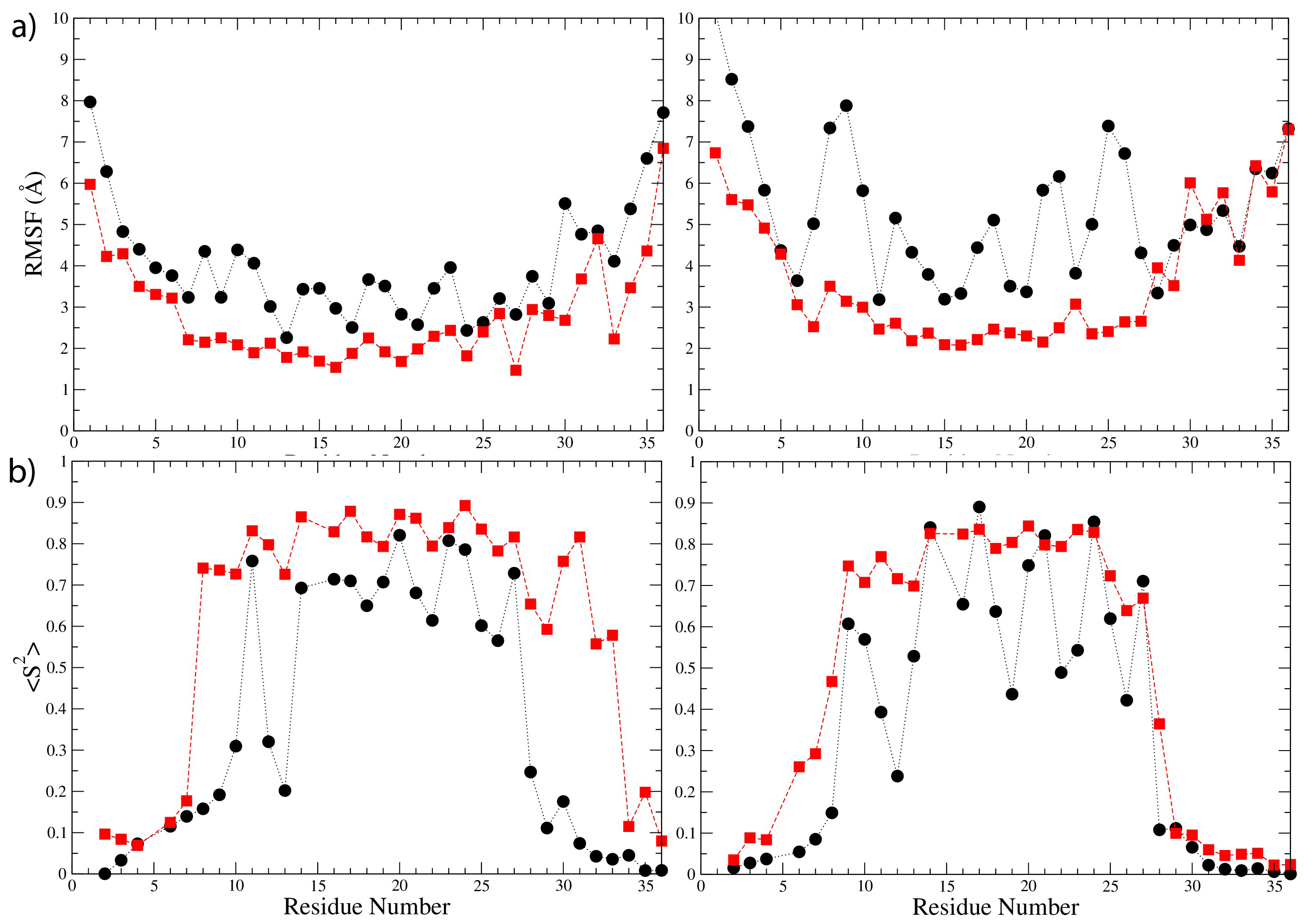
**

**Figure H. RMSF and <S^2^> of Plexin-C1 TM dimers as a function of sequence. a) RMSF and b) <S^2^> of Plexin-C1 TM dimers as a function of sequence for the LH model dimer (Left) and RH model dimer (Right). Data for helix A in black circles, helix B in red squares.**


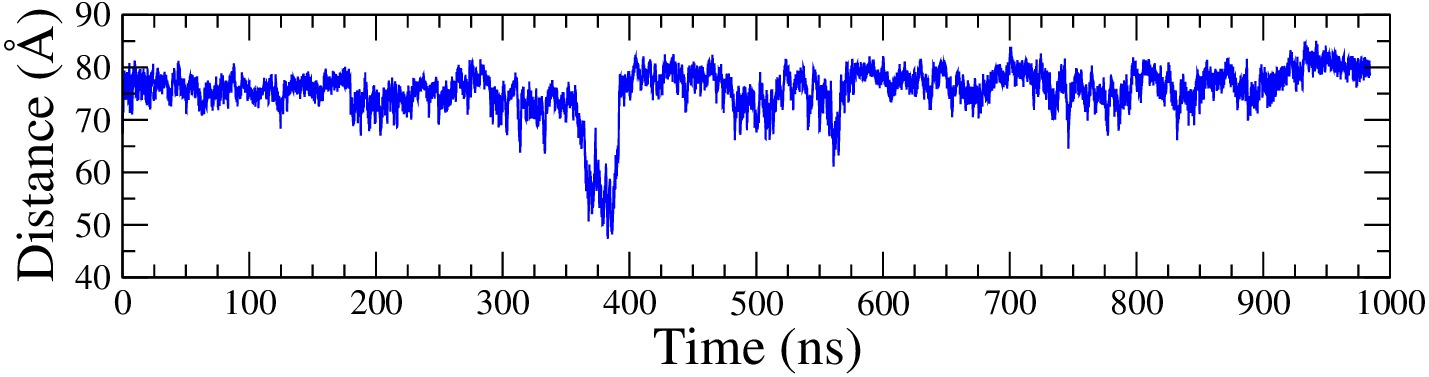


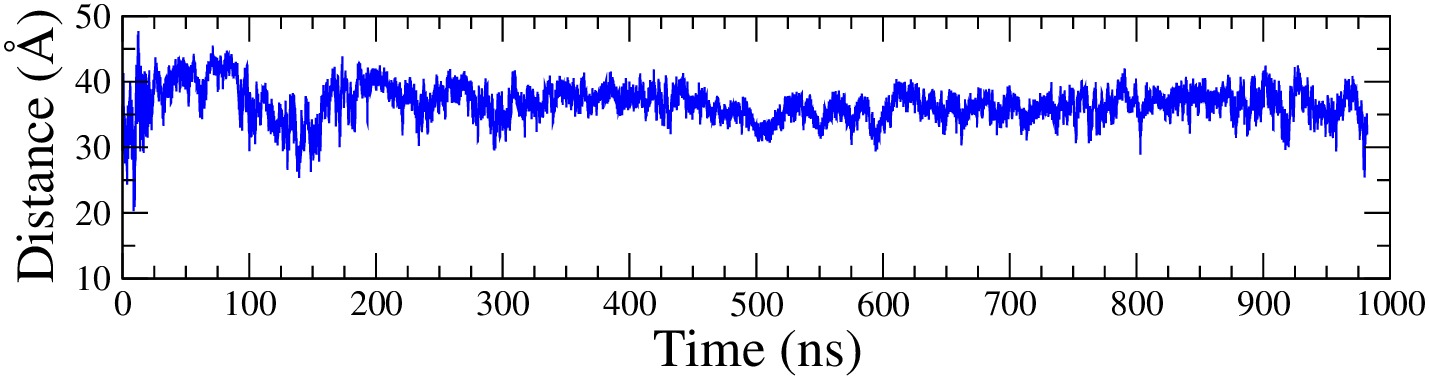


**Figure I.** **Average distances between C-terminal tails (C-alpha of three C-terminal residues) of the JM regions and the inner leaflet of POPC lipid bilayer for plexin-C1 TM-JM dimer in model1/LH model (top) and model2/RH model (Bottom) structures.**

**S1 Table:**

**Table A**. **Full table of PREDDIMER Predictions with Fscore > 2.5.**

| Name | #Model | Fscor | Crossing Angle  (degs) | Rotation-A  (res.4, 11) (degs) | Rotation-B  (res.4, 11) (degs) |
| --- | --- | --- | --- | --- | --- |
| a1 | 1 | 3.0 | -55.1 | 76.6, 68.4 | 93.8 , 89.2 |
|  | 2 | 2.9 | -4.8 | 101.1, 84.4 | 79.4, 60.9 |
|  | 3 | 2.6 | 45.1 | 130.7, 113.8 | 130.7, 113.8 |
|  | 4 | 2.6 | 40.0 | 10.1, -48.3 | 10.1, -48.3 |
| a2 | 1 | 2.9 | -20.1 | -48.7, -70.6 | -48.7, -70.6 |
|  | 2 | 2.7 | -10.4 | -178.9, 146.3 | -178.9, 146.3 |
| a3 | 1 | 2.6 | -5.5 | -166.6, 160.4 | -166.6, 160.4 |
| a4 | 1 | 2.9 | 20.5 | 113.9, 68.2 | 113.9, 68.2 |
|  | 2 | 2.7 | -50.0 | 127.3, 86.5 | 115.5, 74.2 |
| b1 | 1 | 2.9 | -37.0 | 45.6, -3.9 | 4.9, -17.8 |
|  | 2 | 2.8 | -52.1 | 131.2, 166.5 | 120.9, 175.4 |
|  | 3 | 2.6 | 63.2 | -131.8, -175.2 | -154.5, 171.6 |
|  | 4 | 2.7 | 4.9 | 124.9, 108.7 | 152.9, 139.9 |
|  | 5 | 2.7 | 10.5 | -35.6, -39.6 | -35.6, -39.6 |
|  | 6 | 2.7 | -25.1 | 103.4, 87.7 | 103.5, 87.7 |
|  | 7 | 2.6 | -15.4 | 105.0, 71.7 | -24.1, -39.9 |
| b2 | 1 | 3.0 | 60.0 | -145.0, 165.6 | -145.0, 165.6 |
|  | 2 | 2.7 | 55.1 | 74.6, 25.5 | 74.6, 25.5 |
|  | 3 | 2.5 | -35.0 | 106.9, 60.6 | 106.9, 60.6 |
| b3 | 1 | 2.6 | -29.7 | 147.6, 100.8 | -26.6, -79.9 |
|  | 2 | 2.5 | 45.0 | 153.6, 110.9 | 153.6, 110.9 |
|  | 3 | 2.5 | -55.0 | 154.2, 111.4 | 154.2, 111.4 |
| c1 | 1 | 3.4 | 4.9 | -133.3, -93.1 | -133.3, -93.1 |
|  | 2 | 2.5 | -50.0 | -0.49, 30.0 | 6.17, 36.39 |
| d1 | 1 | 3.0 | 55.0 | -98.7 , -150.0 | -104.6, -155.7 |
|  | 2 | 2.6 | -5.1 | -133.0, 176.9 | -118.2, -169.1 |

Note: Similar models were grouped together by RMSD (Table B in S1 File), sequence diversity and also with consideration of crossing angle, followed by similarity of helix rotation, also checking the regions of contact in case of the crossed structures. This reduced the number of models from 26 to 13 (the latter are shown in Table 1).

**Table B. Scaled RMSD between the central regions of initial TM structures from PREDDIMER.** The structures with identifiers in red belong to the groups of 13 selected for further study. The remaining structures (black) are within an RMSD < 3.5 Å close to those selected as shown.


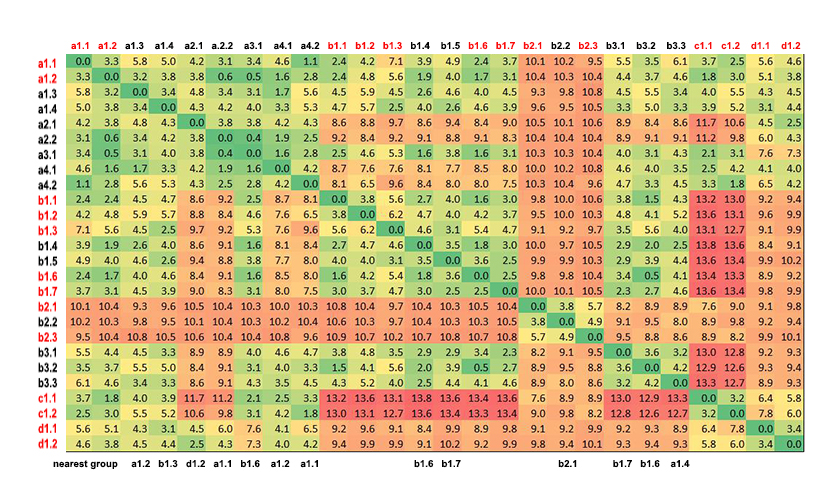


**Table C.** **RMSD, crossing angle, and rotation angles of helices.** **a). RMSD, crossing angle, and rotation angle of helices for plexin-B1 TM trimer model1, TMtimer1+JMmodel1, TM trimer model2, TM trimer2+JMmodel2 after MD simulations.**

| model namefor B1 trimers with/without JM regions | RMSD (Å) | Crossing angle (degs) | Helix A Rotation angle (degs) | Helix B Rotation angle (degs) |
| --- | --- | --- | --- | --- |
| TMmodel1 | 2.6±0.2 | (AB)21.4±3.3 | 6.6±3.6 | 6.6±2.3 |
|  |  | (AC)34.1±3.7 | 13.6±6.7 | 29.0±7.6 |
|  |  | (BC)38.5±3.8 | 7.6±2.6 | 37.0±6.6 |
| TM+JMmodel1 | 3.4±0.1 | (AB)6.0±3.1 | 10.5±4.4 | 11.5±5.3 |
|  |  | (AC)27.1±3.8 | 85.4±34.7 | 139.3±24.6 |
|  |  | (BC)27.1±2.9 | 108.1±40.3 | 128.6±32.1 |
| TMmodel2 | 3.3±0.1 | (AB)20.0±3.9 | 24.2±4.9 | 13.8±5.8 |
|  |  | (AC)20.0±3.7 | 36.6±5.7 | 20.0±7.0 |
|  |  | (BC)26.8±3.9 | 20.9±5.3 | 17.6±3.9 |
| TM+JMmodel2 | 1.9±0.1 | (AB)24.8±3.5 | 48.9±2.9 | 49.7±5.0 |
|  |  | (AC)19.9±2.9 | 47.9±3.5 | 21.2±2.5 |
|  |  | (BC)26.7±4.5 | 37.9±2.5 | 20.0±2.1 |

**b). RMSD, crossing angle, and rotation angles of helices for TM+extension dimers after long-term simulations.**

| Plexin TM dimer model | RMSD (Å) | Crossing angle (degs) | Helix A Rotation angle (degs) | Helix B Rotation angle (degs) |
| --- | --- | --- | --- | --- |
| a1.1 | 2.7 ± 0.5 | 14.2±7.7 | 20.4±9.4 | 16.0±6.7 |
| a1.2 | 3.5±0.2 | 19.0±9.0 | 58.8±39.6 | 86.0±16.3 |
| b1.1 | 2.5±0.2 | 36.9±3.5 | 15.4±4.8 | 19.1±6.8 |
| b1.2 | 4.5±0.4 | 43.9±7.7 | 44.7±13.6 | 37.5±6.9 |
| b1.3 | 4.0±0.4 | 38.5±7.2 | 18.2±7.4 | 14.6±5.0 |
| b1.6 | 3.2±0.5 | 47.0±4.6 | 56.5±13.0 | 17.3±8.2 |
| b1.7 | 5.1±0.9 | 27.0±9.3 | 66.1±12.6 | 20.3±8.1 |
| b2.1 | 13.8±1.4 | 50.0±11.5 | 43.7±18.8 | 94.2±15.3 |
| b2.3 | 4.4±0.5 | 12.9±8.2 | 90.5±16.5 | 34.2±11.2 |
| c1.1 | 3.5±0.4 | 15.5±7.5 | 84.8±24.7 | 32.1±10.7 |
| c1.2 | 3.3±0.4 | 34.6±5.1 | 44.7±5.8 | 32.3±6.0 |
| d1.1 | 3.2±0.2 | 26.2±3.1 | 36.5±6.4 | 19.4±2.9 |
| d1.2 | 3.7±0.3 | 29.6±5.8 | 86.8±8.4 | 29.2±9.1 |

**c). RMSD, Crossing angle and rotation angle for plexin-C1 TM dimers and –C1 TM+JM dimers after MD simulations.**

| Job name | RMSD (Å) | Crossing angle (degs) | Helix A Rotation angle (degs) | Helix B Rotation angle (degs) |
| --- | --- | --- | --- | --- |
| c1.1 | 3.5±0.4 | 15.5±7.5 | 84.8±24.7 | 32.1±10.7 |
| c1.2 | 3.3±0.4 | 34.6±5.1 | 44.7±5.8 | 32.3±6.0 |
| c1TM-JM-model1 | 4.3±0.2 | 13.6±3.5 | 162.4±7.2 | 168.4±6.3 |
| c1TM-JM-model2 | 3.8±0.4 | 70.9±8.9 | 119.4±46.2 | 61.5±25.6 |

**S1 Movie:**

**1. plexin-C1 TM+JM dimer structure showed structure distortion caused by its interaction with lipids during the long term MD simulations, with the initial structure starting from the model1 as shown in Figure 7 Left.**

**2. plexin-C1 TM+JM dimer structure during the long term MD simulations, with the initial structure starting from the model2 as shown in the Figure 7 Right.**
